# Supplementary material for: Neuromorphic hierarchical modular reservoirs
Source: Nat Commun. 2026 Jun 25;17:7962. doi: 10.1038/s41467-026-74466-2 (PMC13448079; doi:10.1038/s41467-026-74466-2)
Supplement: Supplementary file 1 [file 41467_2026_74466_MOESM1_ESM.pdf]

Supplementary Information
Neuromorphic hierarchical modular reservoirs

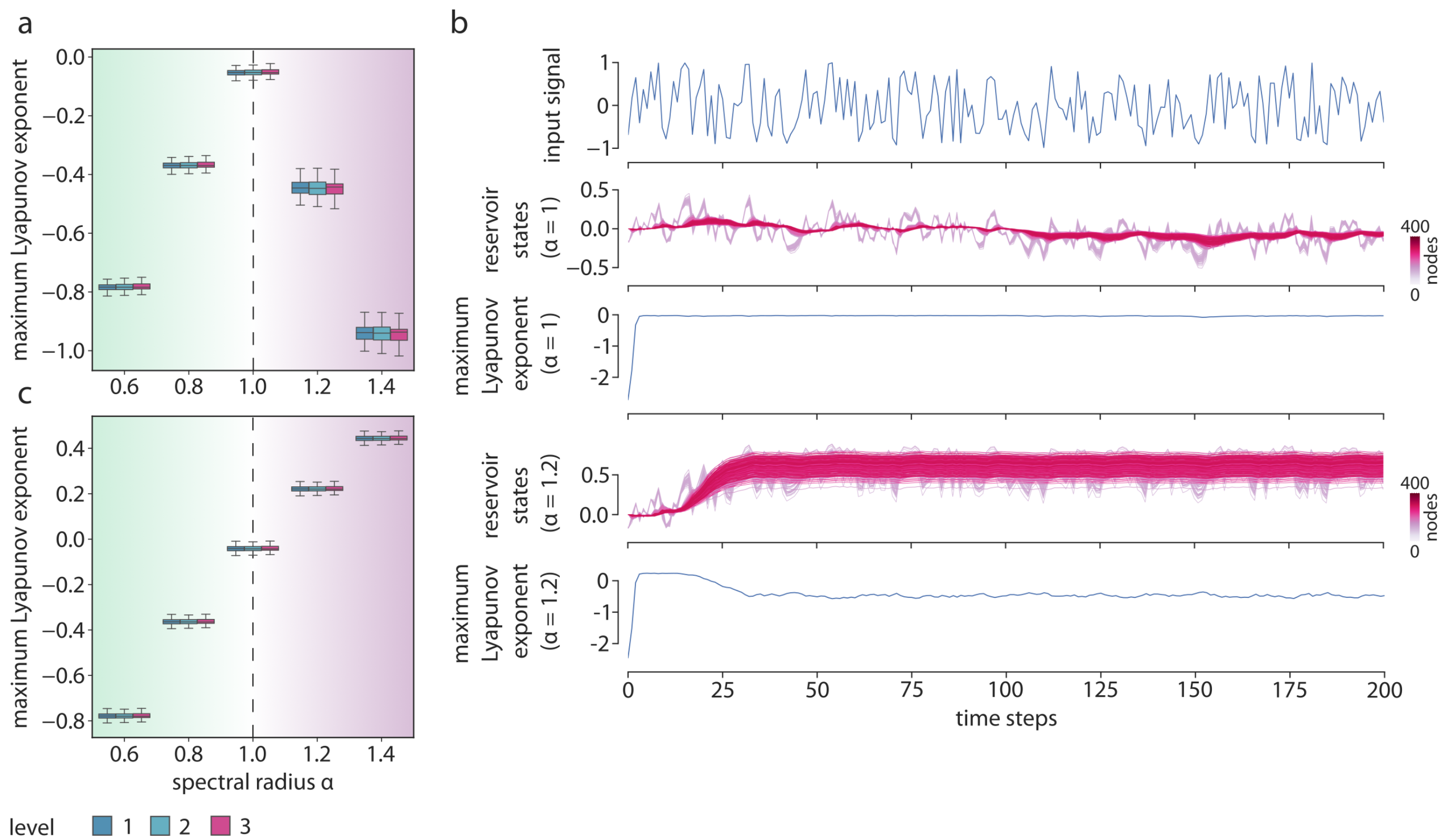

Supplementary Figure 1 **Lyapunov exponent analysis** | (a) Maximum Lyapunov exponent (LE) as a function of the spectral radius α across reservoirs of $\tanh$ units for each hierarchical level. Boxes show the quartiles of the distribution and whiskers extend to the endpoints. Edge-of-chaos criticality is characterized by a negative maximum LE close to zero. Beyond $\alpha = 1$, maximum LE decreases instead of crossing to chaos (positive LE) because of $\tanh$ unit saturation.(b) From top to bottom: Example input signal for the memory capacity task. Reservoir states at $\alpha = 1$ for an example third-level network and the corresponding instantaneous contributions to the maximum Lyapunov exponent (obtained using a QR decomposition algorithm). Reservoir states for the same network at $\alpha = 1.2$ and the corresponding instantaneous contributions to the maximum Lyapunov exponent (notice the positive values prior to saturation). (c) Maximum Lyapunov exponent (LE) as a function of the spectral radius α across linear reservoirs for each hierarchical level. Boxes show the quartiles of the distribution and whiskers extend to the endpoints. Comparatively to the $\tanh$ units, linear units do not saturate, leading to positive maximum LEs consistent with chaos for $\alpha > 1$.

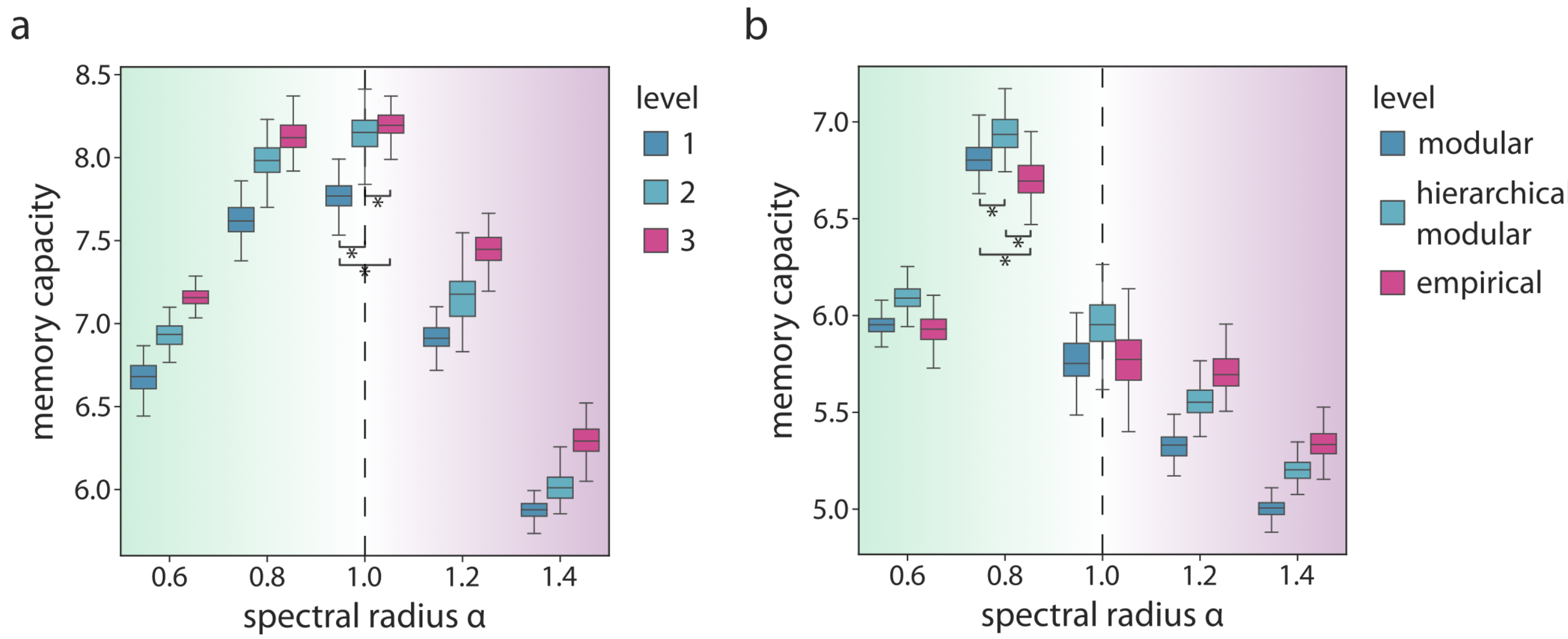

Supplementary Figure 2 **Standard memory capacity** | (a) Same as in Fig. 2b, but with memory capacity measured as the sum of squared Pearson correlation coefficients across all time delays. (b) Same as in Fig. 6c, but with memory capacity measured as the sum of squared Pearson correlation coefficients across all time delays. Asterisks indicate statistical significance of differences between levels according to Wilcoxon-Mann-Whitney tests ($p < 0.01$). Boxes show the quartiles of the distribution and whiskers extend to the endpoints. Significance is only shown for the highest performing regime.

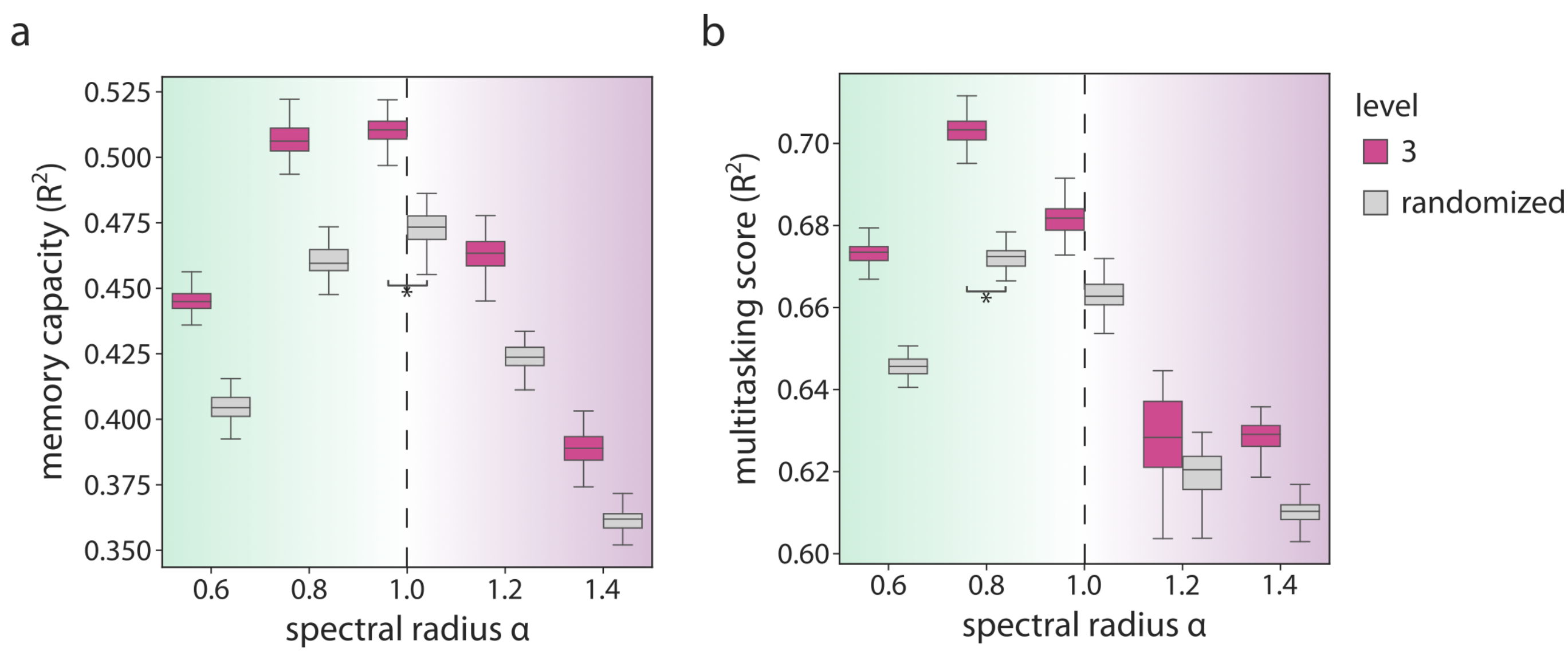

Supplementary Figure 3 **Contrast between hierarchical modular and random reservoir performance** | Memory capacity (a) and multitasking score (b) as a function of the α parameter for level-3 networks and their degree-preserving randomized surrogates. Asterisks indicate statistical significance of differences between levels according to Wilcoxon-Mann-Whitney tests ($p < 10^{-33}$). Boxes show the quartiles of the distribution and whiskers extend to the endpoints. Significance is only shown for the highest performing regime.

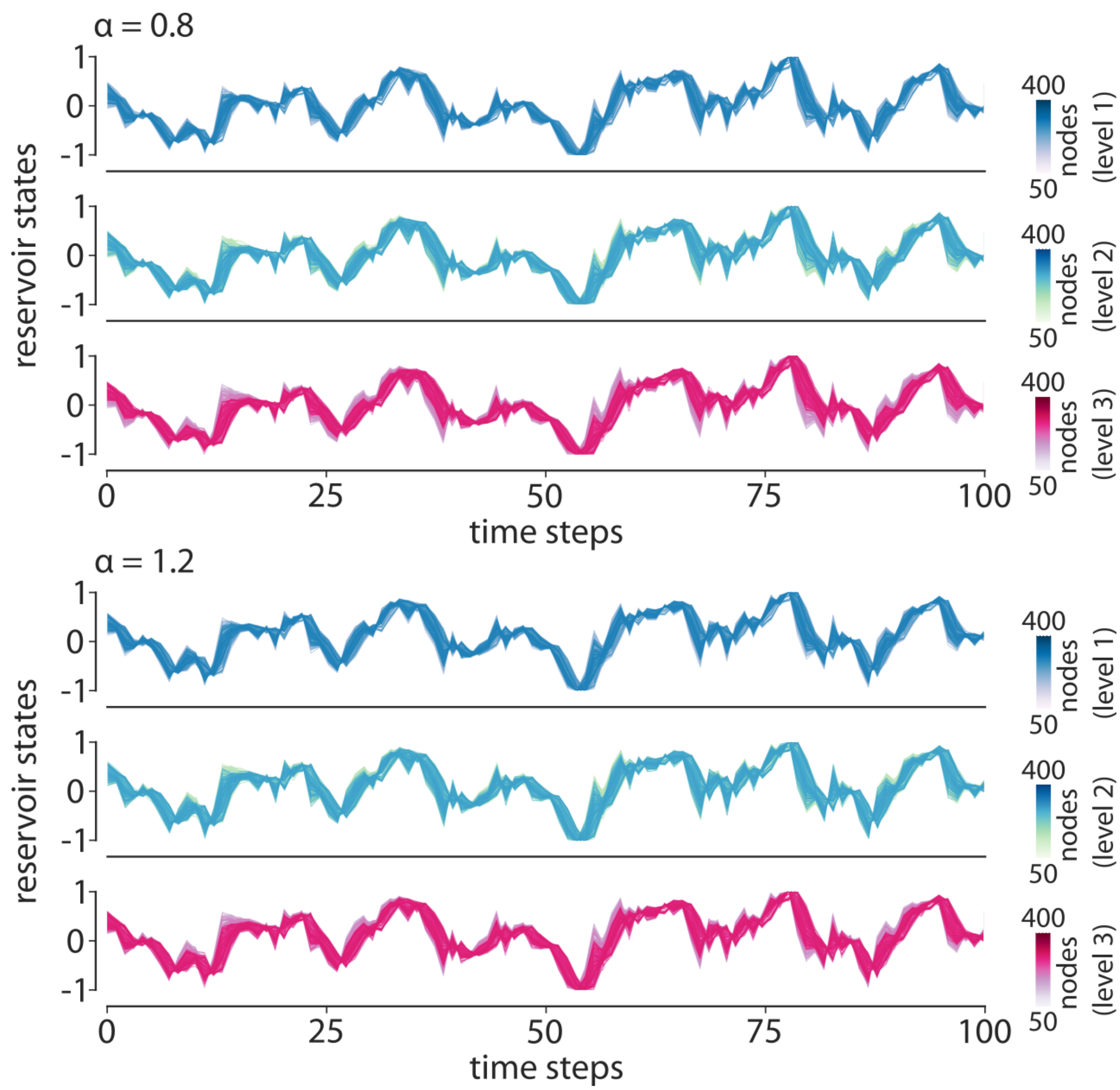

Supplementary Figure 4 **Example reservoir time series** | Output node states at $\alpha = 0.8$ (a) and $\alpha = 1.2$ (b) for an example reservoir of each of the three levels. The time series were rescaled between -1 and 1 to emphasize differences.

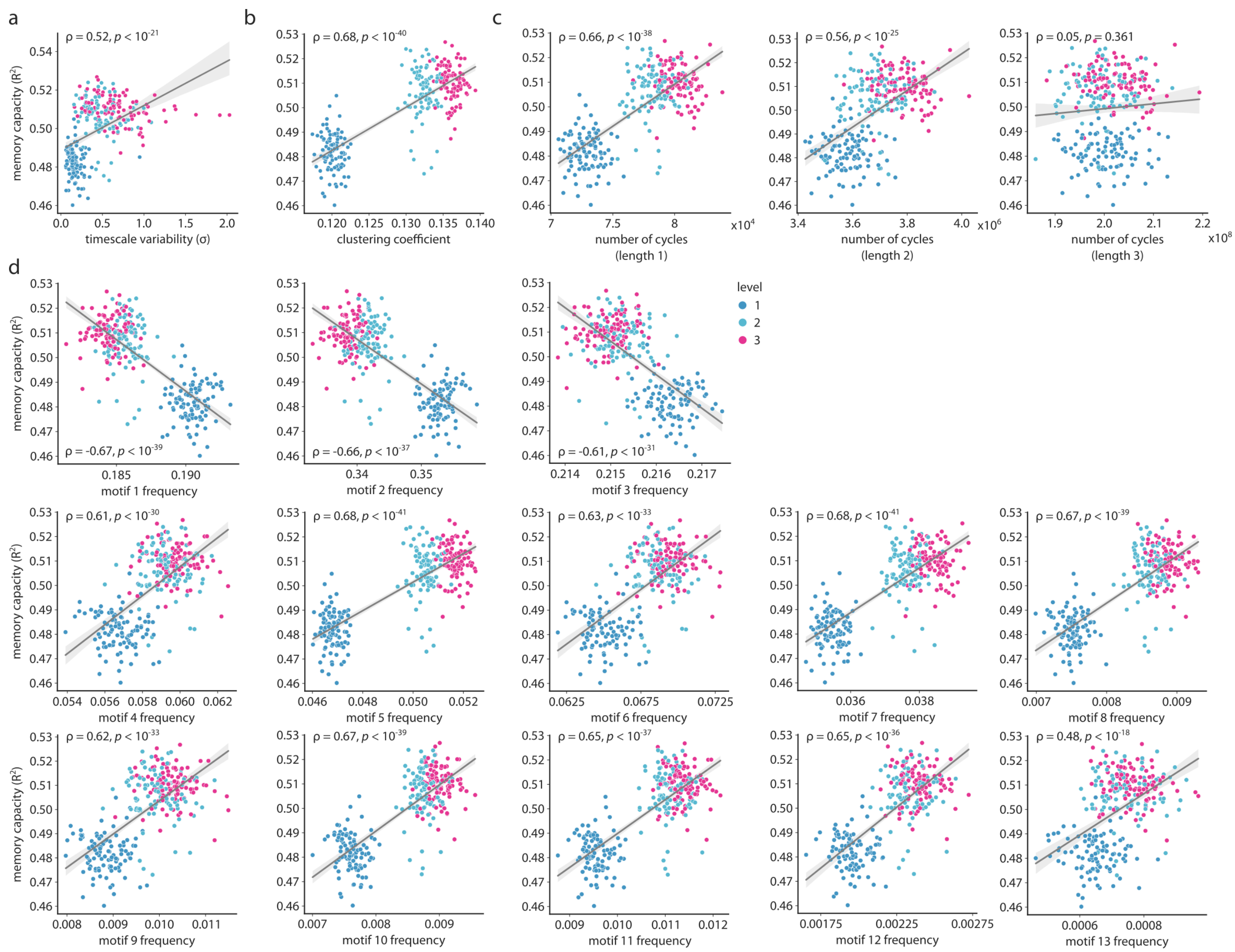

Supplementary Figure 5 **Determinants of (hierarchical) modular reservoir performance** | Relationship between memory capacity ($\boldsymbol{R}^2$) of synthetic (hierarchical) modular reservoirs and their timescale variability (a; in units of standard deviation), clustering coefficient (b), number of cycles of different length (c), and motif frequency (d). Each point represents a network. Linear regression lines and 95% bootstrapped confidence intervals (shaded bands; 1000 samples) are shown for visualization purposes. Spearman correlation coefficients (ρ) and associated p-values are shown in each scatter plot.

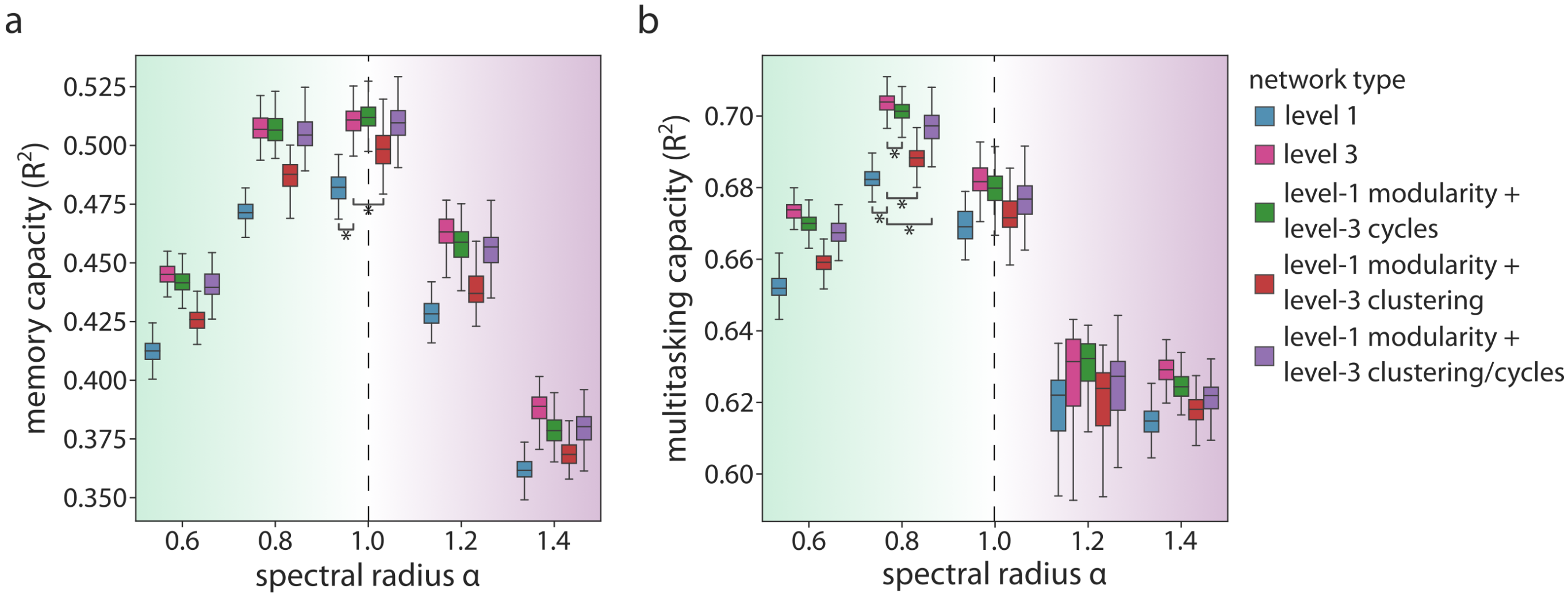

Supplementary Figure 6 **Modularity-, cycles-, and clustering-preserving null models** | Memory capacity (a) and multitasking score (b) as a function of the α parameter for level-1 networks, level-3 networks and modularity-, cycles- and/or clustering-preserving randomized surrogates. Clustering-preserving null networks were generated using the same approach as for cycles-preserving null networks. Asterisks indicate statistical significance of differences between level-3 networks and other network types according to Wilcoxon-Mann-Whitney tests ($p < 10^{-6}$). Boxes show the quartiles of the distribution and whiskers extend to the endpoints. Significance is only shown for the highest performing regime.

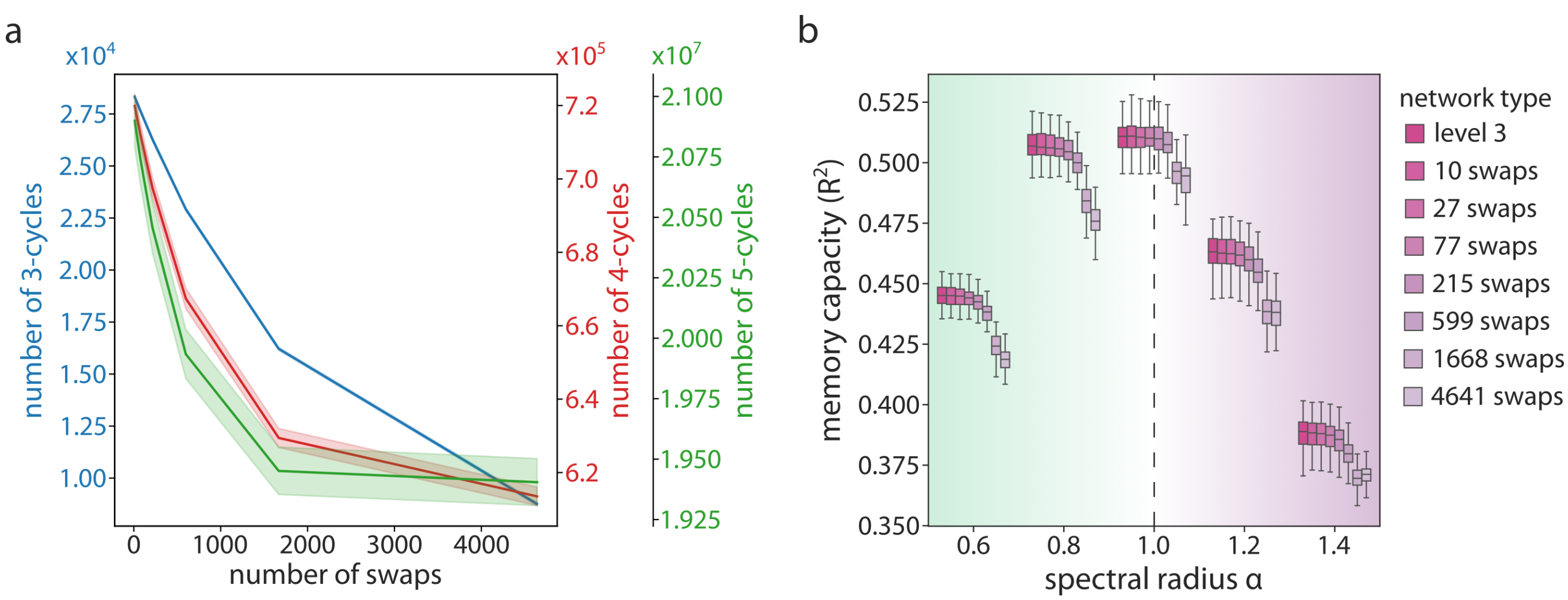

Supplementary Figure 7 **Cycles perturbation experiment** | To remove cycles of length 3-5, we use degree-preserving rewiring with the additional constraint that the double-edge swaps do not create new cycles of length 3. We limit the constraint to 3-cycles as extending it to longer lengths rapidly exhausts valid swaps, preventing meaningful perturbation. Nevertheless, we observe that cycles of lengths 4 and 5 are initially concomitantly destroyed. We stop the procedure when the average number of cycles of any length between 3 and 5 starts increasing. (a) Number of 3-, 4-, and 5-cycles as a function of the number of constrained swaps. Lines represent the mean and shaded bands depict 95% bootstrapped confidence intervals (1000 samples) (b) Memory capacity ($\boldsymbol{R}^2$) as a function of the α parameter for level-3 networks and their rewired surrogates. Boxes show the quartiles of the distribution and whiskers extend to the endpoints.

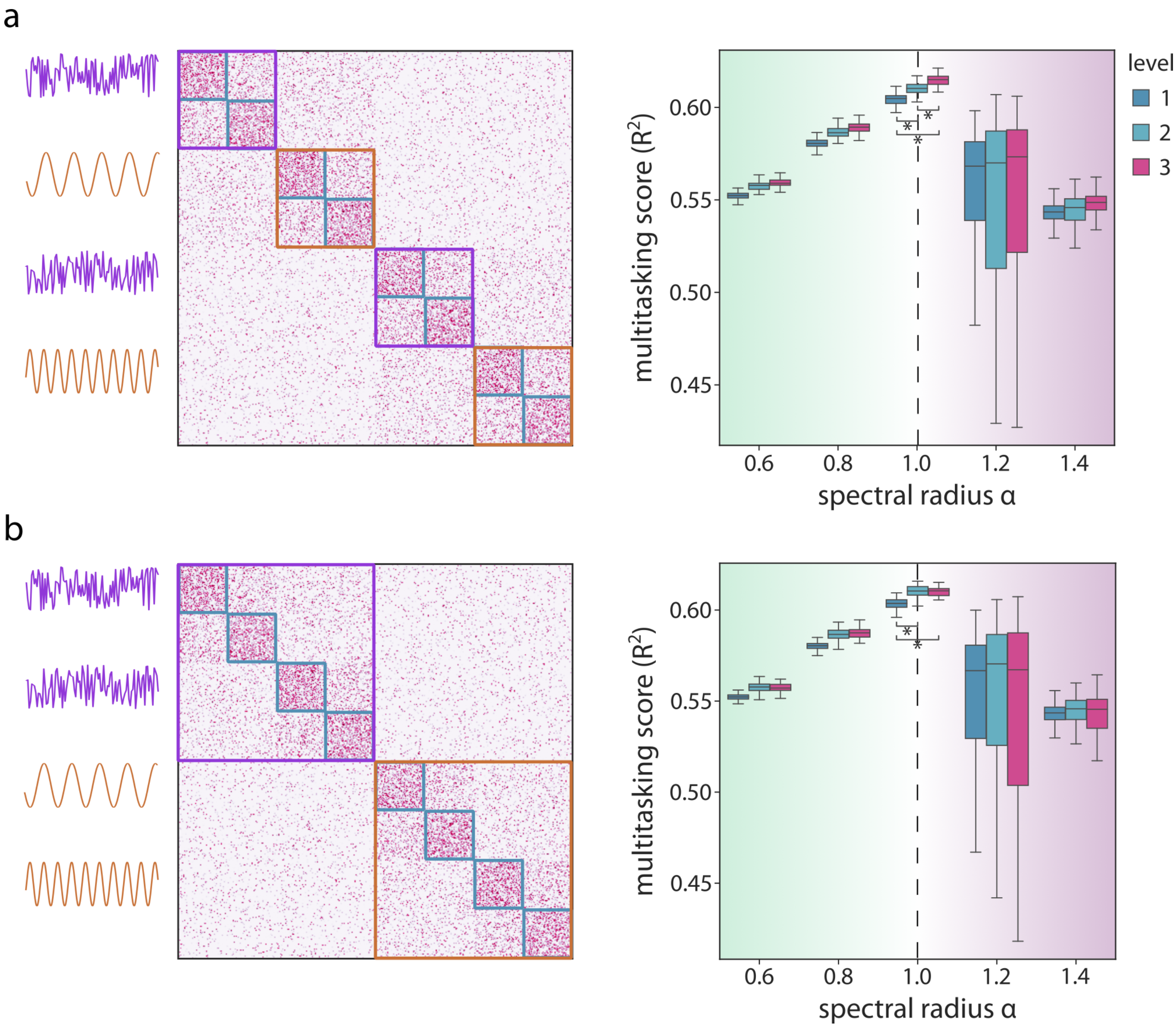

Supplementary Figure 8 **Hierarchical modularity supports multitasking - 4 tasks** | (a) Left: Schematic of the multitasking framework: the 4 second-level modules are each assigned a different task. The memory capacity task and the non-linear transformation task are interleaved. Both input signals are propagated simultaneously across the network. Right: Average $\boldsymbol{R}^2$ regression score across tasks as a function of the α parameter for each hierarchical level. (b) Same as (a) when task categories are not interleaved (the 2 third-level modules are each assigned a different task category). Asterisks indicate statistical significance of differences between levels according to Wilcoxon-Mann-Whitney tests ($p < 10^{-16}$). Boxes show the quartiles of the distribution and whiskers extend to the endpoints. Significance is only shown for the highest performing regime.

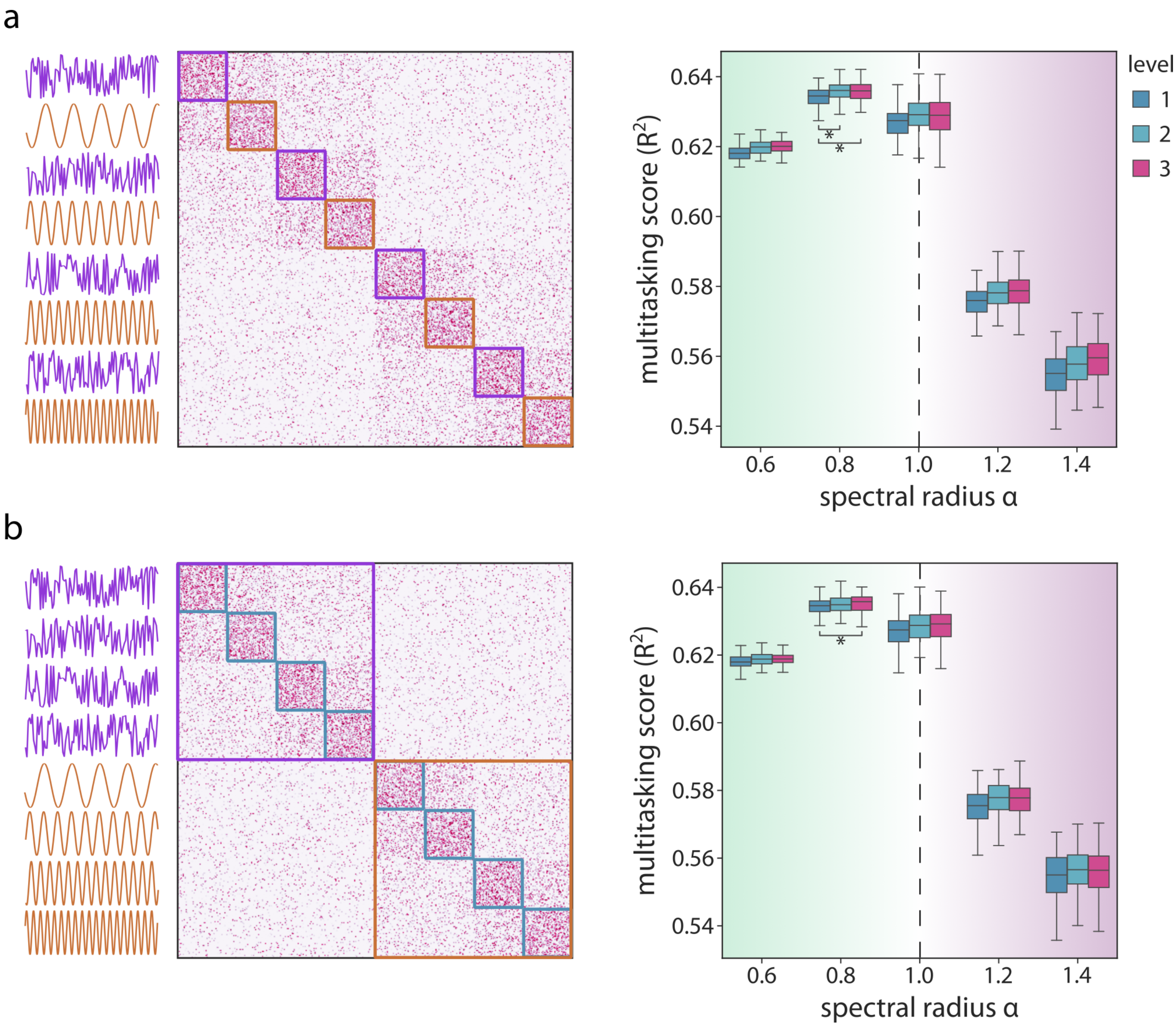

Supplementary Figure 9 **Hierarchical modularity supports multitasking -** 8 **tasks** | (a) Left: Schematic of the multitasking framework: the 8 first-level modules are each assigned a different task. The memory capacity task and the non-linear transformation task are interleaved. Both input signals are propagated simultaneously across the network. Right: Average $\boldsymbol{R}^2$ regression score across tasks as a function of the α parameter for each hierarchical level. (b) Same as (a) when task categories are not interleaved (the 2 third-level modules are each assigned a different task category). Asterisks indicate statistical significance of differences between levels according to Wilcoxon-Mann-Whitney tests ($p < 0.01$). Boxes show the quartiles of the distribution and whiskers extend to the endpoints. Significance is only shown for the highest performing regime.

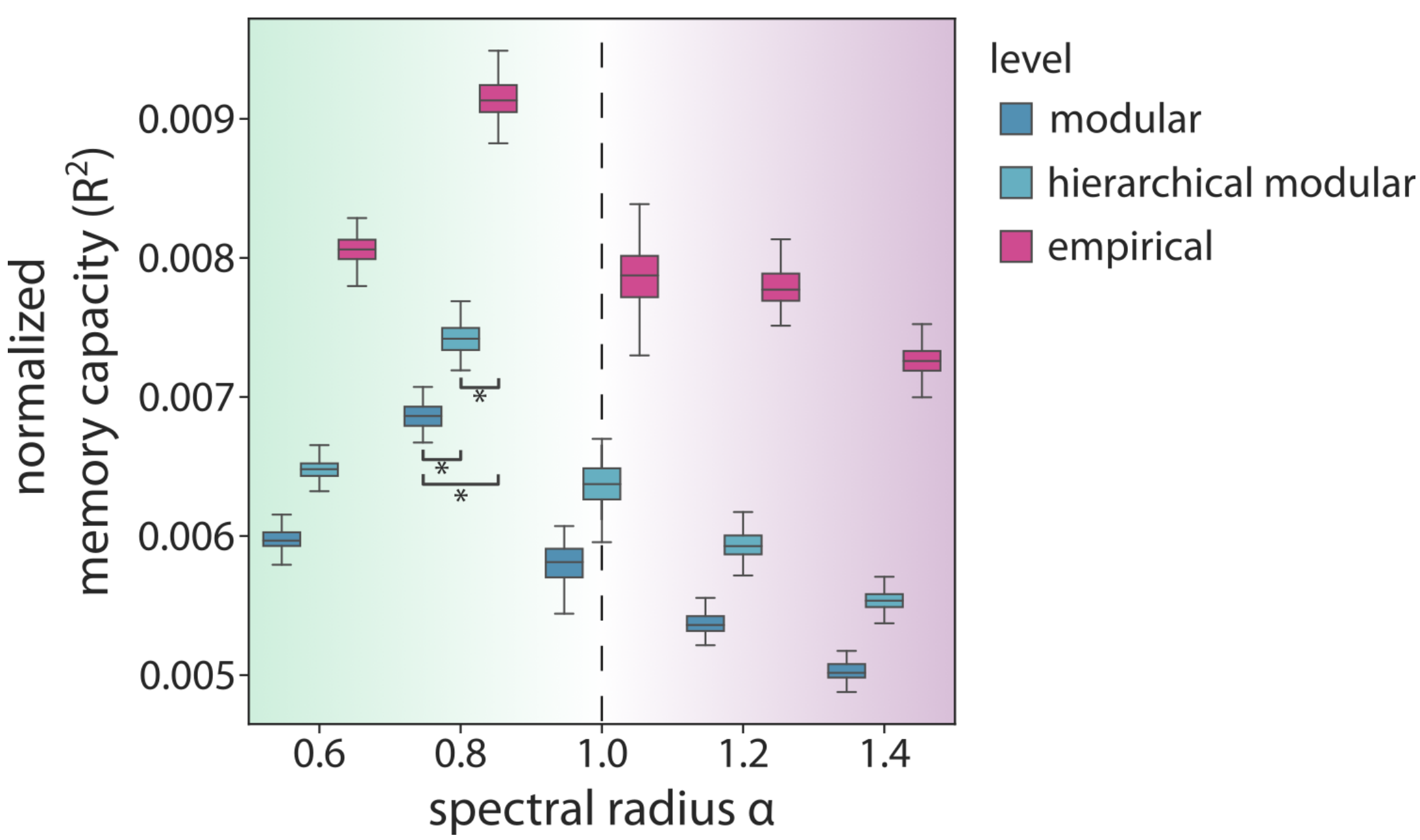

Supplementary Figure 10 **Trade-off between memory capacity and wiring cost in the human connectome** | Memory capacity ($\boldsymbol{R}^2$) normalized by average Euclidean edge length as a function of the α parameter across hierarchical levels. Asterisks indicate statistical significance of differences between levels according to Wilcoxon-Mann-Whitney tests ($p < 10^{-33}$). Boxes show the quartiles of the distribution and whiskers extend to the endpoints. Significance is only shown for the highest performing regime.

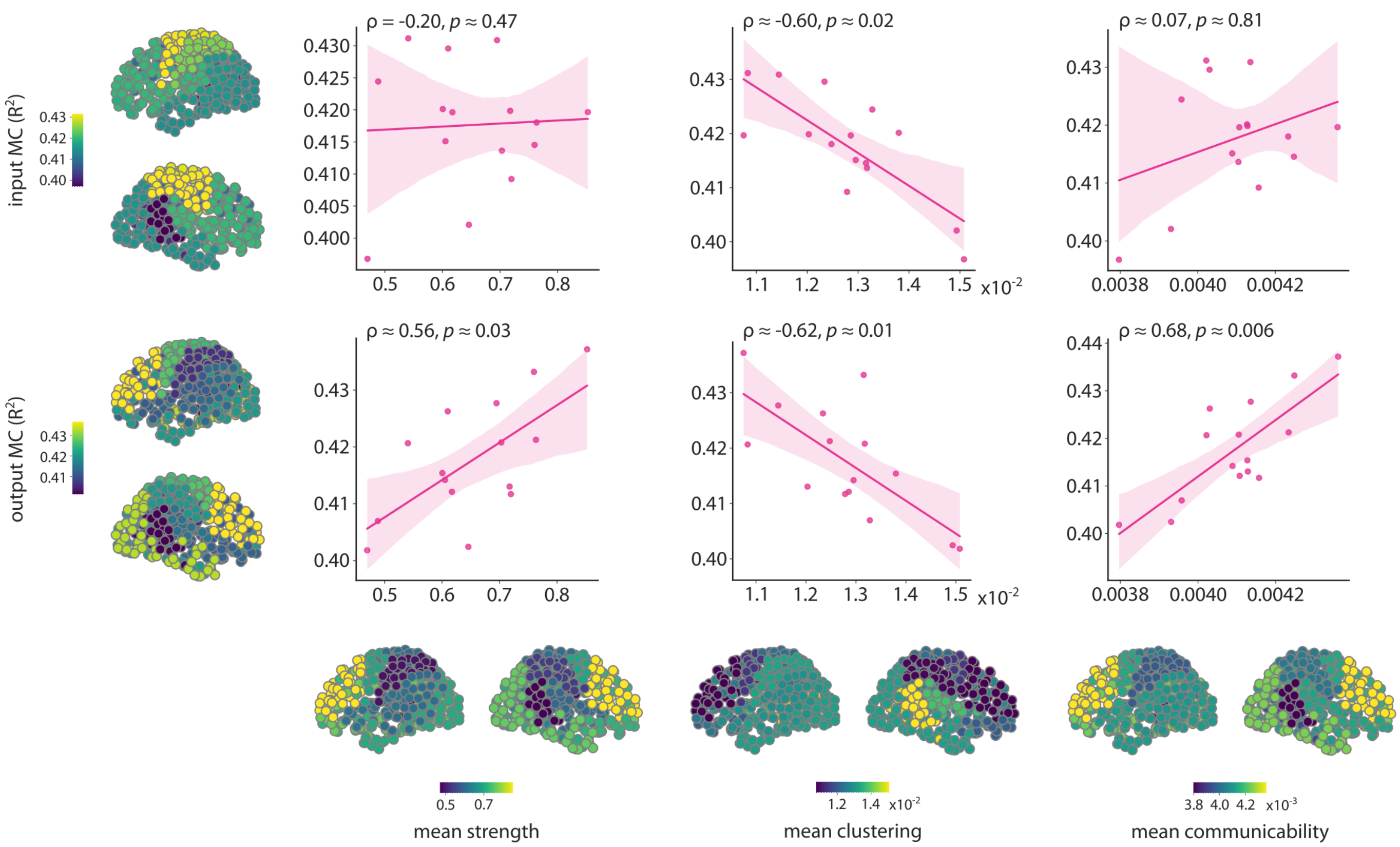

Supplementary Figure 11 **Network determinants of connectome module performance** | Relationships between module-wise memory capacity (MC; $\boldsymbol{R}^2$) as input (top) and output (bottom) as a function of module-average strength (left), clustering (middle), and communicability (right). Each point represents a module. Linear regression lines and 95% bootstrapped confidence intervals (shaded bands; 1000 samples) are shown for visualization purposes. Spearman correlation coefficients (ρ) and associated p-values are shown on top of each scatter plot.

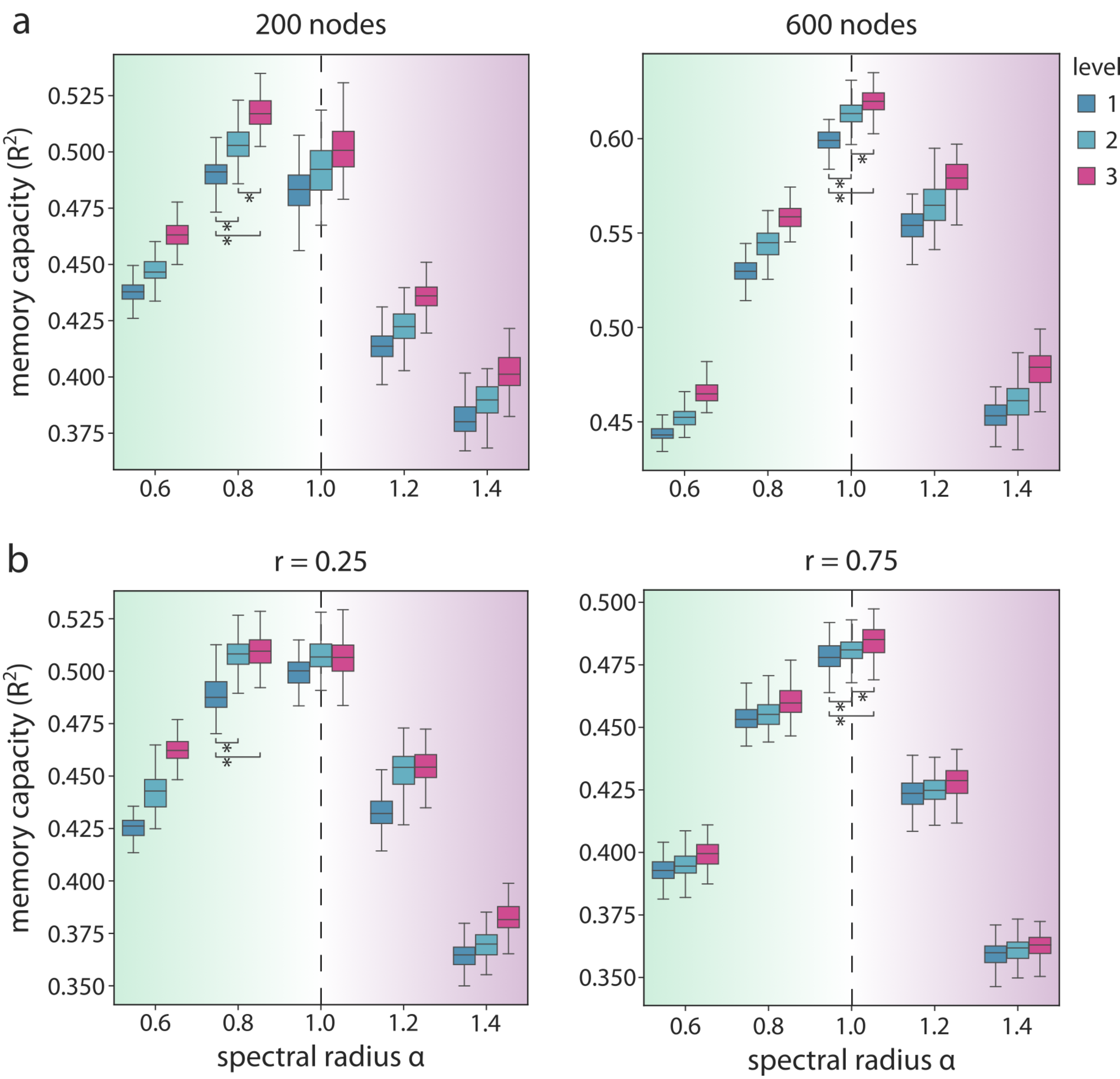

Supplementary Figure 12 **Sensitivity analyses - network size and edge probability scaling** | Memory capacity ($\boldsymbol{R}^2$) as a function of the α parameter across hierarchical levels, for different network sizes (a; 200 and 600 nodes) and different edge probability scaling factors r (b; 0.25 and 0.75). Asterisks indicate statistical significance of differences between levels according to Wilcoxon-Mann-Whitney tests ($p < 0.01$). Boxes show the quartiles of the distribution and whiskers extend to the endpoints. Significance is only shown for the highest performing regime.

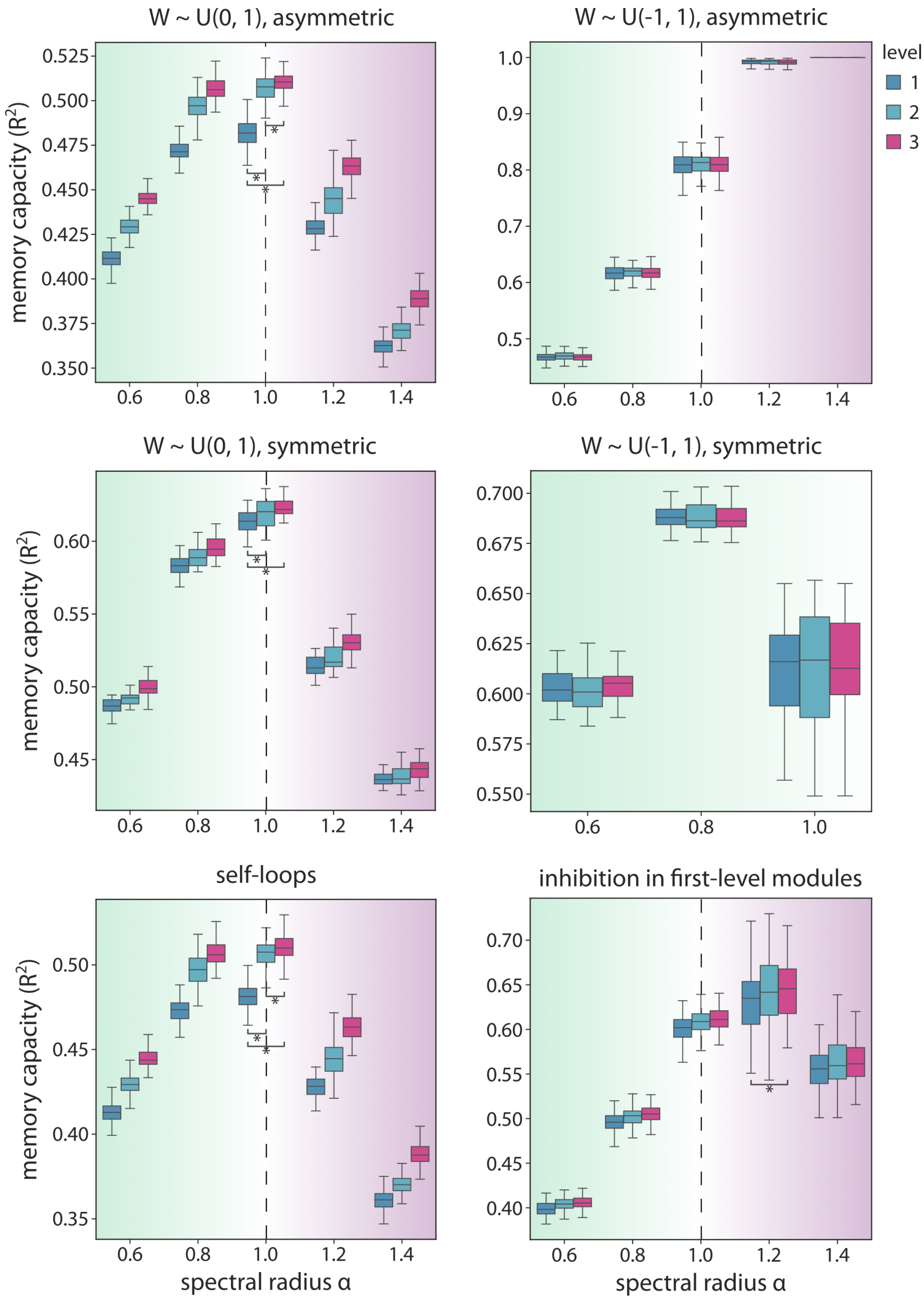

Supplementary Figure 13 **Sensitivity analyses - weight distribution, symmetry, and self-loops** | Memory capacity (R^2) as a function of the α parameter across hierarchical levels, for asymmetric positive networks (top, left), asymmetric signed networks (top, right), symmetric positive networks (middle, left), symmetric signed networks (middle, right), asymmetric positive networks containing self-loops (bottom, left), and asymmetric signed networks only containing negative weights in the first-level modules (bottom, right). Asterisks indicate statistical significance of differences between levels according to Wilcoxon-Mann-Whitney tests ($p < 0.05$). Boxes show the quartiles of the distribution and whiskers extend to the endpoints. Significance is only shown for the highest performing regime. Note that we only show the stable regime for symmetric signed networks for visualization purposes as performance considerably drops in the chaotic regime.

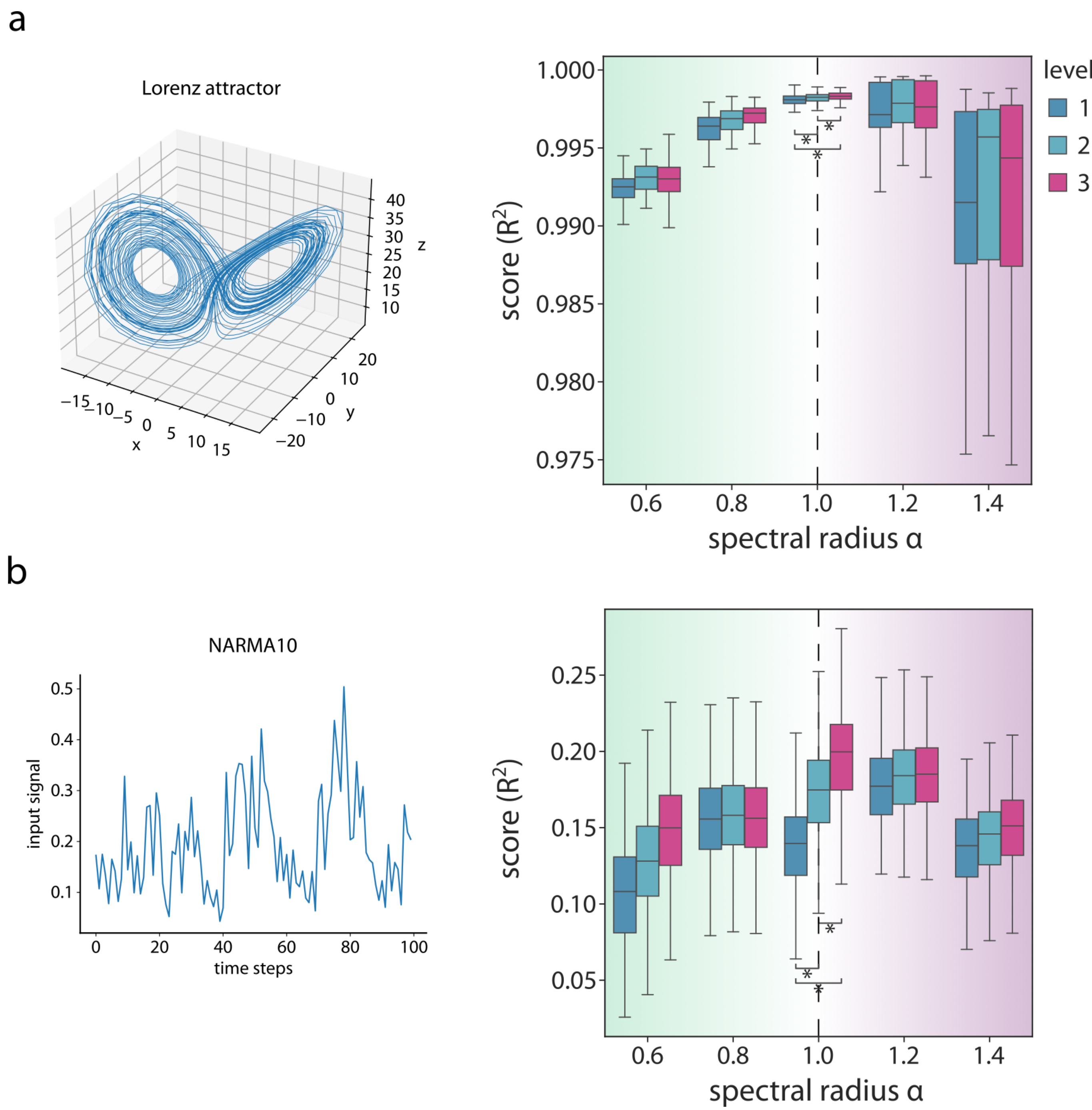

Supplementary Figure 14 **Sensitivity analyses - chaotic time series prediction** | (a) Left: Example Lorenz strange attractor used for the one-step-ahead prediction task. Right: $\boldsymbol{R}^2$ regression score as a function of the α parameter across hierarchical levels. (b) Left: Example NARMA10 time series used for the one-step-ahead prediction task. Right: $\boldsymbol{R}^2$ regression score as a function of the α parameter across hierarchical levels. Asterisks indicate statistical significance of differences between levels according to Wilcoxon-Mann-Whitney tests ($p < 0.05$). Boxes show the quartiles of the distribution and whiskers extend to the endpoints. Significance is only shown for the highest performing regime.
