# Supplementary material for: Neuromorphic hierarchical modular reservoirs
Source: Nat Commun. 2026 Jun 25;17:7962. doi: 10.1038/s41467-026-74466-2 (PMC13448079; doi:10.1038/s41467-026-74466-2)
Supplement: Supplementary file 2 [file 41467_2026_74466_MOESM2_ESM.pdf]

nature portfolio

Peer Review File

Neuromorphic hierarchical modular reservoirs

Corresponding Author: Dr Bratislav Misic

This file contains all reviewer reports in order by version, followed by all author rebuttals in order by version.

Version 0:

Reviewer comments:

Reviewer #1

(Remarks to the Author)
The paper positions itself at the interface of network neuroscience and connectionist AI (reservoir computing in particular). The authors argue that hierarchical modularity (which is a wellknown feature of brain networks, at least in the context of network neuroscience) confers computational advantages when instantiated in recurrent reservoirs. They claim three contributions: (i) a blockmodeling framework that interpolates between flat modular and multilevel hierarchical networks while controlling for size and density, (ii) systematic evaluation of computational capacity (memory, multitasking, temporal diversity) across these architectures, and (iii) a new hierarchical-modularity–preserving null model enabling fair comparison of human connectome data. The broad claim is that hierarchical modularity constitutes a general inductive bias for designing neuromorphic systems and a structural principle underlying neural timescales.

Regarding novelty:
The main new contribution is the focus on hierarchical modularity within a single recurrent graph, rather than modularity alone or layered/stacked reservoirs. The motif/cycle analysis and the development of a hierarchy-preserving null model for connectomic data are additional technical steps that are not standard in prior reservoir comp studies. However, the paper does not make it very clear that hierarchical reservoirs (in their various forms) have already been explored, and those works also reported advantages for memory and multi-scale processing. Thus, while the precise formalization (block-hierarchical SBM + motif analysis + nulls) is new, the conceptual claim that hierarchy benefits learning capacity is not.

Regarding technical depth:
The experiments are well donw and cover synthetic multitasking settingss with connectome-informed networks. The analyses of timescales and motif enrichment provide correlates of improved performance, adding some technical depth. On the other hand, the scope of computational tasks remains limited to memory capacity and simple nonlinear transformations. For a high-impact journal, the work would benefit from either (i) a more ambitious demonstration of how hierarchy supports complex, real-world tasks, or (ii) deeper theoretical results connecting hierarchical structure to specific computational bounds. The current findings are more incremental extensions of modularity results than a paradigm shift.

Some important missing references:
* https://arxiv.org/abs/2105.06923 (Hierarchical Architectures in Reservoir Computing Systems). This is a similar contribution (from 2021) and it is important to contrast methods, metrics, etc.

* https://arxiv.org/pdf/1705.05782 Hierarchical Temporal Representation in Linear Reservoir Computing. This paper supports your “pool of timescales” argument but via depth, not hierarchical modularity. This could be an intereting control comparing graph hierarchy vs layer depth for identical parameter budgets.

* https://arxiv.org/abs/1711.05255 (a paper from 2017 -- Deep-ESN: A Multiple Projection-encoding Hierarchical Reservoir Computing Framework) This has already shown that hierarchical RC improving memory-like abilities.

* https://arxiv.org/abs/2406.06262 (Modular Growth of Hierarchical Networks: Efficient, General, and Robust Curriculum Learning)

Some more specific comments:

* The framing oscillates between “brain-inspired insight” and “neuromorphic engineering.” It is not clear whether the goal is to explain cortical computation, to propose a design principle for AI, or both. This ambiguity dilutes the main contribution.

* Stochastic Block Model construction (p.12, Eq.1–4): The definition assumes perfectly homogeneous modules at each level, with fixed size and edge probabilities. This is an idealized toy model and may artificially amplify “hierarchy effects.” Real connectomes are heterogeneous, yet the claims about “hierarchy per se” are derived mainly from this homogeneous case.

* Hierarchy-removal procedure (Eq.5): Averaging edge probabilities across collapsed levels preserves expected degree but not higher-order correlations. This could bias comparisons between hierarchical vs flat modular networks.

* Signed/negative weights are only addressed briefly in Supplementary Fig. S8, where the effect of hierarchy largely disappears. This raises a major limitation: brain networks are inhibitory–excitatory, and many learning systems require inhibitory edges. The main conclusions of the paper may not be valid when we also consider negative weights.

* Fig2: The comparison shows hierarchical > modular > random. But since modularity strength and hierarchy depth covary in the SBM, it is not clear if the benefit comes from hierarchy per se or just stronger clustering and longer cycles. A control where flat modular networks are matched for clustering and cycle counts is missing.

* Also, the performance difference is statistically significant but effect sizes appear modest (~10–20% gains). For Nature Communications, it must be clarified whether this is functionally meaningful.

* Fig 3 – Timescales:
a) The claim that hierarchical networks yield a “pool of timescales” is based on variability in autocorrelation decay constants. But variability alone does not prove functional benefit unless linked to task performance.
b) The timescale measure is highly sensitive to noise and spectral radius scaling. No robustness checks with alternative definitions (e.g., exponential fit vs spectral entropy, power-law fits) are presented.

* Fig. 4: Motif enrichment is reported but not normalized against randomized graphs that preserve motif distributions at lower levels. Without motif-specific nulls, the interpretation that “reciprocal/cyclic motifs drive memory” is speculative.

The link between cycles and information storage is cited but no direct task-based manipulation (e.g., remove cycles of length 3–5 and retest memory capacity) is attempted.

* Fig. 5: The multitasking framework is adapted from Loeffler et al. 2021. However, only one multitask configuration is shown prominently; others are relegated to Supplementary (S4, S5). The key issue is whether hierarchy provides robust advantage across all multitask scenarios. In Fig. S5 (8 tasks), third-level hierarchy gives no extra improvement over two levels, raising doubt that “deeper hierarchy” adds real benefit.

* Fig 6: The hierarchical null model is a methodological contribution. However, it is unclear if it preserves other important structural statistics (edge length distribution, degree assortativity). If not, the observed differences may reflect confounds.

* The result that null networks outperform empirical connectomes in stable regimes ($\alpha < 1$) is brushed aside as “other topological features are detrimental.” This undermines the biomimetic argument: why should the real brain underperform synthetic surrogates if hierarchical modularity is its key advantage?

* Timescale comparison to MEG data shows a correlation $\rho \approx 0.44$, which is statistically strong but moderate. The null distributions (Fig. 6d right) show that hierarchical nulls achieve similar correlations, so hierarchy alone cannot explain empirical timescales.

Overall assessment:
In my opinion the paper offers a carefully executed demonstration that hierarchical modularity improves reservoir comp performance, but its big picture contribution is less significant than presented. The novelty lies in formalizing hierarchy within SBM reservoirs and introducing nulls, rather than in the central idea that hierarchy supports memory and multitasking. The work is solid, but whether it rises to the level of Nature Communications depends on how the editors weigh methodological rigor against conceptual novelty. Without clearer differentiation from prior hierarchical RC work and without more challenging demonstrations, the contribution risks being seen as incremental.

(Remarks on code availability)

Reviewer #2

(Remarks to the Author)
This manuscript reports results of a new mathematical model that investigates the effects of hierarchically organised modular networks on learning and memory capacity, studied using reservoir computing (RC). While all the results are based on simulation, the authors have integrated empirical human connectome data from MEG. The results are original and noteworthy and presented well overall. Below I provide some comments and suggested edits that I hope will serve to improve the manuscript.

- Methods: Details about the sizes of the modular networks (including the number of output nodes used in the RC tasks) should be mentioned upfront. Some details are only mentioned in sensitivity analysis and in Fig. S7. The number of nodes & edges is important for reproducibility and benchmarking, and also since it determines RC performance.

- Figs. 2b, 5b & 6c (and supp figs): I suggest removing the title "criticality" since it may be misinterpreted and is anyway not needed in the figures. As the authors state themselves, dynamics are sometimes referred to as critical at alpha=1, but the descriptor criticality could easily be misinterpreted by some neuroscience readers as avalanche criticality, which would be incorrect as no activity propagation is shown. Edge of stability or edge of chaos criticality is OK to make this distinction - see e.g. Kanders et al. "Avalanche and edge-of-chaos criticality do not necessarily co-occur in neural networks." Chaos: An Interdisciplinary Journal of Nonlinear Science 27.4 (2017); and Hochstetter et al. "Avalanches and edge-of-chaos learning in neuromorphic nanowire networks." Nature Communications. 12, 4008 (2021).

- Fig. 2b & 6c: MC results are shown as R^2, but it would be helpful to show the raw MC values, since this is what is usually reported in RC studies and would make comparisons & benchmarking much easier. These could be supplementary figures. Note that the MC task was originally introduced to evaluate fading memory due to recurrence in RNNs, so a note should be made in relation to Fig. 4 that shows the enhanced recurrent motifs and number of cycles in higher levels as corroborating/explaining Fig.2b.

- Fig. 3b: can you please also provide 2 additional supplementary figures showing corresponding output node states for alpha<1 and alpha >1.

- Fig. 4: can you add a legend at the bottom right and also assuming this is for alpha=1 networks, add that to the caption.

- Multitasking results: these are very interesting. Are you able to provide further explanation for why the more complex multitasking scenarios in Figs. S4 & S5 show performance peaking at alpha=1, whereas Fig. 5b shows the peak at alpha=0.8? Also, did you try to regress to a different waveform to check whether eoc criticality only benefits the more computationally complex task of regressing to a square wave (as in Hochstetter et al.)? Note that the two multitasking tasks in Loeffler et al. were selected because they operate on different dynamical timescales: MC is fast, whereas waveform regression is relatively slow, and they leverage different resources - recurrent loops vs. internal dynamics. Here, hierarchical modularity seems to contribute to both. This might be worth mentioning if the authors find it interesting.

- Discussion: the overall results showing how hierarchical modularity can enhance learning and memory by expanding timescale diversity is very interesting. But the connectome results also show that topology can't explain everything and that better dynamical models (more realistic than tanh) should be considered. One suggestion I have (for future work) which would be relatively easy to implement is to consider adding internal memory to the nodes - see e.g. Verstraeten et al. An experimental unification of reservoir computing, Neural Networks, 20, 391 (2007).

- typo, p.10: motif consists in --> motif consists of

(Remarks on code availability)
I did not install or run the code, but it appears to be complete and includes a README file with clear instructions, data details and additional information on external dependencies.

Version 1:

Reviewer comments:

Reviewer #1

(Remarks to the Author)
The revised manuscript represents a clear and substantive improvement over the original submission. I would like to thank the authors for their thorough rebuttal and for revising the paper significantly.

The paper has been strengthened by 1. expanding the experimental scope to include more challenging benchmarks such as Lorenz and NARMA tasks, 2. providing a much more convincing mechanistic account through the introduction of cycle-preserving null models and direct perturbation experiments, which clarify the role of recurrent motifs in driving memory capacity, and 3) improving the robustness and interpretability of their analyses, particularly for neural timescales. The additional connectome-based analyses and the consideration of wiring cost tradeoffs also enhance the biological relevance of the study.

At the same time, some of my earlier concerns remain: The overall conceptual novelty is still somewhat limited relative to prior work on hierarchical and modular reservoir architectures, and the practical significance of the observed performance gains remains modest. Moreover, the results suggest that much of the benefit attributed to hierarchical modularity may in fact be explained by lower order structural features such as cycles, raising questions about how essential hierarchy itself is as a design principle. Finally, certain limitations (such as the sensitivity to the treatment of negative weights and the reliance on relatively stylized tasks) indicate that further work is needed to establish the generality and practical applicability of the findings.

(Remarks on code availability)

Reviewer #2

(Remarks to the Author)
The authors have comprehensively responded to my comments and I'm glad they had an opportunity to correct their code. I have no further comments or suggestions.

(Remarks on code availability)
I reviewed the additional tasks they have now implemented.

Open Access This Peer Review File is licensed under a Creative Commons Attribution 4.0 International License, which permits use, sharing, adaptation, distribution and reproduction in any medium or format, as long as you give appropriate credit to the original author(s) and the source, provide a link to the Creative Commons license, and indicate if changes were made.
In cases where reviewers are anonymous, credit should be given to 'Anonymous Referee' and the source.
The images or other third party material in this Peer Review File are included in the article's Creative Commons license, unless indicated otherwise in a credit line to the material. If material is not included in the article's Creative Commons license and your intended use is not permitted by statutory regulation or exceeds the permitted use, you will need to obtain permission directly from the copyright holder.
To view a copy of this license, visit https://creativecommons.org/licenses/by/4.0/

Dear Reviewers,

Thank you for the constructive feedback on our first submission, and for the opportunity to revise the manuscript. Following your comments and suggestions, we have thoroughly revised the manuscript. In this letter, we respond to each of the reviewers' comments in detail. Reviewer comments are in **bold font** and our responses are in regular font.

Major changes include:

- We clarify the objective of the paper as a study of the function of hierarchical modularity in neural networks to avoid ambiguity.
- We show that hierarchical modularity can yield significant improvements in chaotic time-series prediction tasks.
- We create a novel modularity-, cycles-, and clustering-preserving randomization algorithm and implement perturbation experiments targeting short cycles to confirm their role in the benefits of hierarchical modularity for memory capacity.
- We confirm the robustness of our timescale measure by extending our analyses to the complementary measure of spectral entropy.
- We show that the empirical connectome outperforms (hierarchical) modularity-preserving nulls when accounting for average connection length. This finding reinforces the notion that the brain balances an economic trade-off between functionality and cost of its topological organization.
- We show that the advantages of hierarchical modularity for multitasking are robust to changes in the nonlinear transformation task.

Reviewer #1 (Remarks to the Author):

The paper positions itself at the interface of network neuroscience and connectionist AI (reservoir computing in particular). The authors argue that hierarchical modularity (which is a wellknown feature of brain networks, at least in the context of network neuroscience) confers computational advantages when instantiated in recurrent reservoirs. They claim three contributions: (i) a blockmodeling framework that interpolates between flat modular and multilevel hierarchical networks while controlling for size and density, (ii) systematic evaluation of computational capacity (memory, multitasking, temporal diversity) across these architectures, and (iii) a new hierarchical-modularity–preserving null model enabling fair comparison of human connectome data. The broad claim is that hierarchical modularity constitutes a general inductive bias for designing neuromorphic systems and a structural principle underlying neural timescales.

Regarding novelty:
The main new contribution is the focus on hierarchical modularity within a single recurrent graph, rather than modularity alone or layered/stacked reservoirs. The motif/cycle analysis and the development of a hierarchy-preserving null model for connectomic data are additional technical steps that are not standard in prior reservoir comp studies. However, the paper does not make it very clear that hierarchical reservoirs (in their various forms) have already been explored, and those works also reported advantages for memory and multi-scale processing. Thus, while the precise formalization (block-hierarchical SBM + motif analysis + nulls) is new, the conceptual claim that hierarchy benefits learning capacity is not.

We thank the Reviewer for their comment, which allows us to make better contact with the wider literature (see below). We agree with the Reviewer that both modularity and hierarchy have already been studied as architectural principles for reservoir computing (RC), but we believe that hierarchical modularity (multiple levels of nested modules) goes beyond a simple combination of modularity and hierarchy as it was operationalized in previous RC studies, i.e., layers of reservoirs involving sequential processing. Furthermore, it is an important organizational principle of brain networks. As such, and as observed by the Reviewer, we extend our study of hierarchical modularity to empirical connectivity patterns of the human brain using a new hierarchical modularity-preserving null. Altogether, this represents a fundamentally new contribution focused on studying the computational function of brain-like hierarchical modularity patterns.

Regarding technical depth:
The experiments are well donw and cover synthetic multitasking settingss with connectome-informed networks. The analyses of timescales and motif enrichment provide correlates of improved performance, adding some technical depth. On the other hand, the scope of computational tasks remains limited to memory capacity and simple nonlinear transformations. For a high-impact journal, the work would benefit from either (i) a more ambitious demonstration of how hierarchy supports complex, real-world tasks, or (ii) deeper theoretical results connecting hierarchical structure to specific computational bounds. The current findings are more incremental extensions of modularity results than a paradigm shift.

We thank the Reviewer for acknowledging the quality of our experiments and for suggesting ways to add technical depth. Following the Reviewer's recommendation, we now additionally demonstrate the computational advantage of hierarchical modularity in two chaotic time series prediction tasks, derived from the widely used Lorenz and NARMA systems. We choose the Lorenz task because it models a real-world weather system (atmospheric convection) and the NARMA task because it combines nonlinearity and memory, making it very complex. For both tasks, peak performance is observed at the edge of chaos ($\alpha = 1$), with higher-order hierarchical modular networks systematically outperforming their lower-order counterparts ($p < 0.05$, for all two-tailed, Wilcoxon–Mann–Whitney two-sample rank-sum tests). Interestingly, effect sizes are much larger for the more difficult NARMA task (common-language effect size (CLES) = 68.72-88.34%) than for the Lorenz task (CLES = 58.99-68.61%), perhaps reflecting a ceiling effect. Altogether, these results show that the computational advantages of hierarchical modularity can extend to more complex tasks, reinforcing its potential as a versatile neuromorphic design principle. Below, we detail the addition of these experiments to the manuscript:

("Results" section, "Sensitivity analyses", Paragraph #2):

> "Next, we go beyond architecture modeling choices and extend our sensitivity analyses to more complex chaotic system prediction tasks commonly used as reservoir computing benchmarks. Specifically, we use the Lorenz task, which models a real-world weather system, and the NARMA task, which combines nonlinearity and memory, making it highly complex. We implement both tasks as one-step-ahead prediction tasks (see *Methods*). For both tasks, peak performance is achieved at the edge of chaos ($\alpha = 1$), with higher-order hierarchical modular networks systematically outperforming their lower-order counterparts (Fig. S14, $p < 0.05$, $CLES \geq 58.99\%$, for all two-tailed,

Wilcoxon–Mann–Whitney two-sample rank-sum tests). These results show that the computational advantages of hierarchical modularity can extend to more complex tasks, reinforcing its potential as a versatile neuromorphic design principle for artificial neural networks.”

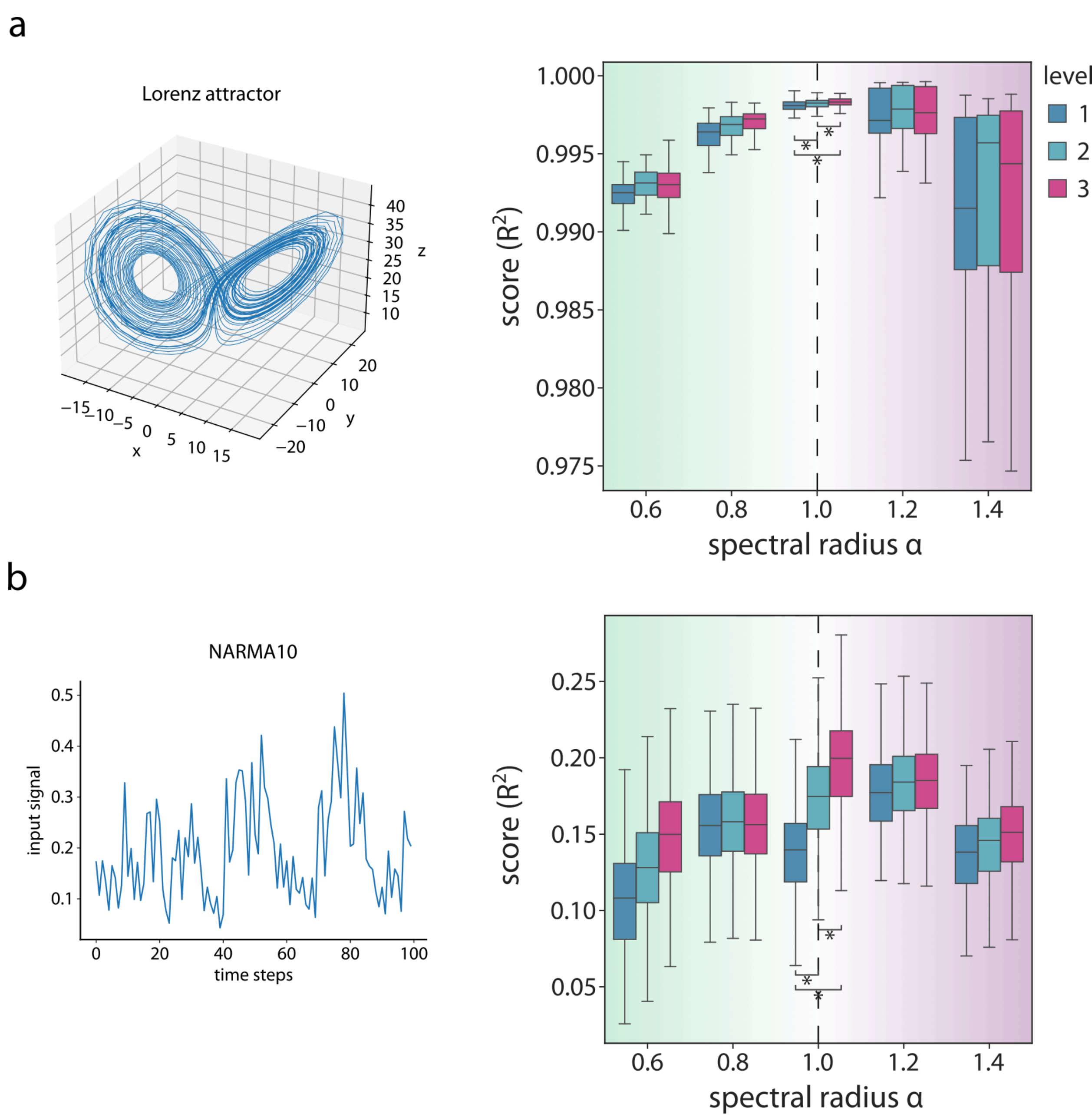

Figure S14 **Sensitivity analyses - chaotic time series prediction** | (a) Left: Example Lorenz strange attractor used for the one-step-ahead prediction task. Right: R^2 regression score as a function of the α parameter across hierarchical levels. (b) Left: Example NARMA10 time series used for the

one-step-ahead prediction task. Right: R^2 regression score as a function of the α parameter across hierarchical levels. Asterisks indicate statistical significance of differences between levels according to Wilcoxon-Mann-Whitney tests ($p < 0.05$). Significance is only shown for the highest performing regime.

("Methods" section, "Dynamical systems"):

"To evaluate chaotic time series prediction, we use two standard reservoir computing tasks: the Lorenz (Lorenz, 1963; Lukoševicius, 2007; Ma et al., 2023; Nadiga, 2021; Smith et al., 2022) and the NARMA10 tasks (Atiya & Parlos, 2000; Inubushi & Yoshimura, 2017, Jaeger, 2002; Kubota et al., 2021; Rodan & Tino, 2010; Verstraeten et al., 2007). We chose the Lorenz task because it models a real-world weather system, i.e., atmospheric convection, and the NARMA task because it combines nonlinearity and memory, making it highly complex. Both tasks were implemented as one-step-ahead prediction tasks and performance was measured using the $\mathbf{R}^2$ coefficient of determination.

As for the memory capacity task, we separately generated a training and a testing input signal of 2100 time points each and a warmup period of 100 time points was discarded from the resulting reservoir state trajectories. The signals were generated using the reservoirpy open-source package (Trouvain et al., 2020; https://reservoirpy.readthedocs.io/en/stable/index.html). Different signals were also used for each network evaluated. An input module was randomly selected among the 8 first-level modules. One input node per input signal was then randomly selected from the input module. All the 350 nodes in all the other 7 modules were used as output. A single readout module was trained for all outputs.

Lorenz

The state (x, y, z) of the Lorenz system evolves according to the following set of three coupled nonlinear differential equations:

$$\frac{dx}{dt} = \sigma(y - x)$$
$$\frac{dy}{dt} = x(\rho - z) - y$$
$$\frac{dz}{dt} = xy - \beta z,$$

with standard parametrization $(\sigma = 10,\ \beta = 8/3,\ \rho = 28)$ for chaotic behavior. The system state is initialized following a uniform distribution in [-20, 20] for each variable and the time series are approximated using a Runge-Kutta method of order 5(4) with time step $\delta t = 0.03t$.

NARMA

The nonlinear autoregressive moving average (NARMA) system is defined on discrete time. Let the state and input of the system at time *t* be $\boldsymbol{y_t}$ and $\boldsymbol{u_t}$, respectively. The tenth-order NARMA system is expressed by:

$$y_{t+1} = \alpha y_t + \beta y_t \sum_{i=0}^{9} y_{t-1} + \gamma u_t u_{t-9} + \delta$$

Following previous work (Inubushi & Yoshimura, 2017, Jaeger, 2002; Kubota et al., 2021; Rodan & Tino, 2010; Verstraeten et al., 2007), parameters ($\alpha, \beta, \gamma, \delta$) are set to (0.3, 0.05, 1.5, 0.1). The system state is initialized at 0 and the input signal follows a uniform distribution in [0, 0.45] (Kubota et al., 2021)."

Atiya, A. F., & Parlos, A. G. (2000). New results on recurrent network training: unifying the algorithms and accelerating convergence. *IEEE transactions on neural networks*, *11*(3), 697-709.

Inubushi, M., & Yoshimura, K. (2017). Reservoir computing beyond memory-nonlinearity trade-off. *Scientific reports*, *7*(1), 10199.

Jaeger, H. (2002). Adaptive nonlinear system identification with echo state networks. *Advances in neural information processing systems*, *15*.

Kubota, T., Takahashi, H., & Nakajima, K. (2021). Unifying framework for information processing in stochastically driven dynamical systems. *Physical Review Research*, *3*(4), 043135.

Lorenz, E. N. (1963). Deterministic Nonperiodic Flow. *Journal of Atmospheric Sciences*, *20*(2), 130-141.

Lukoševicius, M. (2007). Echo state networks with trained feedbacks. *Networks*, *4*.

Ma, H., Prosperino, D., & Räth, C. (2023). A novel approach to minimal reservoir computing. *Scientific Reports*, *13*(1), 12970.

Nadiga, B. T. (2021). Reservoir computing as a tool for climate predictability studies. *Journal of Advances in Modeling Earth Systems*, *13*(4), e2020MS002290.

Rodan, A., & Tino, P. (2010). Minimum complexity echo state network. *IEEE transactions on neural networks*, *22*(1), 131-144.

Smith, L. M., Kim, J. Z., Lu, Z., & Bassett, D. S. (2022). Learning continuous chaotic attractors with a reservoir computer. *Chaos: An Interdisciplinary Journal of Nonlinear Science*, *32*(1).

Trouvain, N., Pedrelli, L., Dinh, T. T., & Hinaut, X. (2020, September). Reservoirpy: an efficient and user-friendly library to design echo state networks. In *International Conference on Artificial Neural Networks* (pp. 494-505). Cham: Springer International Publishing.

Verstraeten, D., Schrauwen, B., d'Haene, M., & Stroobandt, D. (2007). An experimental unification of reservoir computing methods. *Neural networks*, *20*(3), 391-403.

Some important missing references:
*** https://arxiv.org/abs/2105.06923 (Hierarchical Architectures in Reservoir Computing Systems). This is a similar contribution (from 2021) and it is important to contrast methods, metrics, etc.**

*** https://arxiv.org/pdf/1705.05782 Hierarchical Temporal Representation in Linear Reservoir Computing. This paper supports your "pool of timescales" argument but via depth, not hierarchical modularity. This could be an intereting control comparing graph hierarchy vs layer depth for identical parameter budgets.**

*** https://arxiv.org/abs/1711.05255 (a paper from 2017 -- Deep-ESN: A Multiple Projection-encoding Hierarchical Reservoir Computing Framework) This has already shown that hierarchical RC improving memory-like abilities.**

*** https://arxiv.org/abs/2406.06262 (Modular Growth of Hierarchical Networks: Efficient, General, and Robust Curriculum Learning)**

We have now integrated these references in the *Introduction*, acknowledging the literature on hierarchical reservoir computing architectures, and explaining how our approach advances that field:

("Introduction", Paragraphs #1-2):

> "Modularity is a fundamental principle in neuroscience, shaping our understanding of neural architecture, dynamics, and function. The brain is not a homogeneous system, but a complex network, composed of distinct yet interacting modules. In network neuroscience, modularity is a defining feature of brain organization, manifesting in the segregation of structural and functional subnetworks that support specialized processing (Chen et al., 2008; He et al., 2009; Meunier et al., 2010; Sporns & Betzel, 2016). These notions echo a deeply rooted principle in cognitive science, which suggests that cognitive processes operate within informationally encapsulated domains (Coltheart, 1999; Fodor, 1983), highlighting modularity as a key characteristic of mental architecture. More recent perspectives suggest that cognition emerges from a hierarchy of increasingly polyfunctional nested circuits (Betzel & Bassett, 2017; Hilgetag & Goulas, 2020; Meunier et al., 2009, 2010; Park & Friston, 2013), dynamically recruited to support both domain-specific and integrative processes (Anderson et al., 2013; Shine & Poldrack, 2018). Thus, understanding modularity is essential not only for characterizing the brain's structural and functional topology but also for elucidating the computational principles that underlie human cognition.
>
> Beyond neurobiological organization, modularity is also increasingly studied in artificial intelligence (Sun & Guyon, 2023), both as a network feature (Achterberg et al., 2023; Clune et al., 2013; Csordas et al., 2021; Filan et al., 2021; Gu et al., 2024; Lange et al., 2022; Liu et al., 2023; Malakarjun et al., 2023; Tanner et al., 2023) and as a design principle (Amer & Maul, 2019; Andreas et al., 2016; Chang et al., 2018; Goyal et al., 2021; Kirsch et al., 2018; Mittal et al., 2022; Pfeiffer et al., 2023; Purushwalkam et al., 2019; Rosenbaum et al., 2019). In this context, a module can consist of any neural architecture component, from a layer to a whole network as part of an ensemble (Fedus et al., 2022; Jacobs et al., 1991; Pfeiffer et al., 2023; Sun & Goyon, 2023). In deep learning, modular systems leverage intrinsic or imposed modularity in data and tasks via routing functions to select relevant modules and aggregation functions to combine their outputs (Pfeiffer et al., 2023; Sun et al., 2023). Such approaches have been shown to offer key advantages such as enhanced interpretability (Kirsch et al., 2018; Liu et al.,

> 2023; Purushwalkam et al., 2019), efficiency (Andreas et al., 2016; Fedus et al., 2022; Mittal et al., 2022; Pfeiffer et al., 2023; Shazeer et al., 2017), and generalization (Andreas et al., 2016; Chandra et al., 2016; Chang et al., 2018; Kirsch et al., 2018; Mittal et al., 2022; Purushwalkam et al., 2019; Schug et al., 2024), enabling systems to decompose complex tasks and recombine learned solutions (Andreas et al., 2016; Chang et al., 2018; Kirsch et al., 2018; Liu et al., 2023; Purushwalkam et al., 2019; Rosenbaum et al., 2019; Schug et al., 2024). Related ideas also appear in reservoir computing, where modular or hierarchical architectures, such as deep echo state networks, stack multiple reservoirs to enable sequential processing and generate multiscale temporal representations (Gallicchio et al., 2017; Hamidi et al., 2024; Ma et al., 2017; Moon & Lu, 2021). In contrast, the present work investigates topological modularity and hierarchy in a single recurrent neural network."

Some more specific comments:

*** The framing oscillates between "brain-inspired insight" and "neuromorphic engineering." It is not clear whether the goal is to explain cortical computation, to propose a design principle for AI, or both. This ambiguity dilutes the main contribution.**

We thank the Reviewer for their constructive criticism. Our main goal is to study the function of hierarchical modularity in neural networks. While hierarchical modularity is a defining feature of brain organization, we use artificial neural networks, and more specifically reservoir computing, as a modeling tool, because it allows us to mechanistically relate network structure, dynamics, and function. Our results can therefore inform us on structure-function relationships in both artificial (directly) and biological (indirectly) neural networks. This is why we interpret the functional advantage of hierarchical modularity in our experiments both as a potential explanation of its presence in the brain, and as a potential inductive bias for RNN design. However, we do not want to give the impression that the goal of the paper is to propose a new state-of-the-art reservoir architecture. We therefore clarify our approach in the *Introduction* and emphasize the limitations of hierarchical modularity as a design principle in the *Results*:

("Introduction", Paragraph #4):

> "... Only the readout module is trained, allowing us to constrain the network with arbitrary connectivity patterns that remain unchanged throughout learning. Furthermore, since the dynamics of the reservoir are constrained

by its fixed wiring, they can be tuned and maintained by the experimenter (Suárez et al., 2021). This allows the exploration of the interplay between structure, dynamics, and cognitive function."

("Introduction", Paragraph #5):

"... Our results offer new insights on structure-function relationships in artificial neural networks and provide a potential explanation for the presence of hierarchical modularity in biological neural networks."

("Results" section, "Sensitivity analyses", Paragraph #1):

"… However, no significant difference was found between levels of the modularity hierarchy when including negative weights ($\boldsymbol{W} \sim U(-1, 1)$). Furthermore, asymmetric networks including negative weights can achieve near perfect performance at high $\boldsymbol{\alpha}$ values. Importantly, note that the goal of this paper is not to present hierarchical modularity as a new state-of-the-art reservoir architecture, but to study its function in various modeling frameworks. In line with this objective, we take a more biomimetic approach to negative weight placement, constraining them to local within-module connections. Interestingly, this approach restores the effect of hierarchical modularity, despite a drop in performance compared to allowing negative weights throughout the network. Altogether, these results confirm that the computational advantages of hierarchical modularity are robust to numerous modeling settings."

*** Stochastic Block Model construction (p.12, Eq.1–4): The definition assumes perfectly homogeneous modules at each level, with fixed size and edge probabilities. This is an idealized toy model and may artificially amplify "hierarchy effects." Real connectomes are heterogeneous, yet the claims about "hierarchy per se" are derived mainly from this homogeneous case.**

We agree with the Reviewer that our hierarchical modular network model is a simplified representation of the hierarchical modular architecture of the brain. We use it because it provides a controlled environment for studying hierarchical modularity, allowing us to parametrically tune the number of hierarchical levels, the number of modules at each level, as well as their prominence (modularity). It also isolates a nested modular hierarchy from other network properties such as degree heterogeneity.

Nevertheless, our ultimate objective is to understand how hierarchical modularity shapes cognitive function in the brain. This is why in our final analysis, we use empirical structural connectivity patterns of the human brain, identify nested modules of varying size and density, and develop a novel network null model that allows us to separate the effects of modularity and hierarchical modularity. Importantly, these null models preserve empirical heterogeneity in module size and density, allowing us to robustly characterize the advantage of heterogeneous hierarchical modularity from an empirical human connectome.

(“Results” section, “Generating hierarchical modular reservoirs”, Paragraph #1):

> “... A straightforward method for implementing such networks is stochastic blockmodeling (see *Methods*), which allows us to parametrically tune the number of hierarchical levels, the number of modules at each level, as well as their prominence (modularity). To facilitate systematic comparison between networks with different numbers of hierarchical levels, we develop a method to iteratively remove levels of hierarchy, while preserving basic network features, such as network size, density, degree, and modularity. This ultimately yields a continuum from strictly modular networks to hierarchical modular networks.”

(“Results” section, “Hierarchical modularity in the human connectome”, Paragraph #1):

> “Until now, we have considered synthetic blockmodel graphs that are ostensibly neuromorphic, in so far as they reflect the multi-scale nested organization of brain modules (Bassett et al., 2010, Betzel et al., 2024). However, these networks lack the heterogeneity of empirical brain connectomes, that is, real-world modules vary in size and in the number of sub-modules that they contain. To account for these differences, we endow reservoirs with empirical structural connectivity patterns of the human brain. … To isolate the effect of hierarchical modularity on the performance of connectome-informed reservoirs, we develop a novel hierarchical modular network null model.”

*** Hierarchy-removal procedure (Eq.5): Averaging edge probabilities across collapsed levels preserves expected degree but not higher-order correlations. This could bias comparisons between hierarchical vs flat modular networks.**

We believe that averaging edge probabilities across collapsed levels is the most principled way to control network size and expected density, degree, and modularity in

our stochastic block models. Importantly, most null models used in network science do not go beyond preserving degree (Orsini et al., 2015; Váša & Mišić, 2022). It is not immediately clear what higher-order graph features would covary with hierarchical depth or how one could control for them across hierarchical levels without introducing additional biases. Numerous higher-order features might naturally arise from hierarchical modularity without having been explicitly encoded in the generation process. These “spandrels” should not necessarily be controlled for as they are inextricably linked to the architecture under study (Rubinov, 2016). However, isolating them can inform us on the specificity of an effect on network function. This is why in our response to the Reviewer’s next comment, we develop a novel network randomization algorithm that preserves degree, modularity, but also cycle counts, a higher-order structure.

Orsini, C., Dankulov, M. M., Colomer-de-Simón, P., Jamakovic, A., Mahadevan, P., Vahdat, A., ... & Krioukov, D. (2015). Quantifying randomness in real networks. *Nature communications*, *6*(1), 8627.

Rubinov, M. (2016). Constraints and spandrels of interareal connectomes. *Nature communications*, *7*(1), 13812.

Váša, F., & Mišić, B. (2022). Null models in network neuroscience. *Nature Reviews Neuroscience*, *23*(8), 493-504.

*** Fig2: The comparison shows hierarchical > modular > random. But since modularity strength and hierarchy depth covary in the SBM, it is not clear if the benefit comes from hierarchy per se or just stronger clustering and longer cycles. A control where flat modular networks are matched for clustering and cycle counts is missing.**

We thank the Reviewer for this suggestion. To answer this question, we develop a novel network null model building on the degree and modularity-preserving approach applied to empirical connectomes, but additionally preserving average clustering and cycle counts using simulated annealing. We find that the increase in the number of cycles of lengths 3 and 4 in hierarchical modular networks is sufficient to explain their superior memory capacity relative to their strictly modular counterparts. While this indicates that the improvement is not specific to a hierarchical modular architecture, it nevertheless informs us on the mechanisms underlying its computational advantage. It also positions our hierarchical modular network model as a principled approach for introducing cycles of different lengths in a modular network. Furthermore, we find that the multitasking performance of hierarchical modular networks remains superior to that of the degree,

modularity, and cycles-preserving nulls. This reinforces the idea that a hierarchical modular architecture can allow advantageous input and output placement for multitasking, beyond the effect of lower-order features such as clustering and cycles. Below, we detail the addition of these findings to the manuscript:

(“Results” section, “Hierarchical modularity generates a pool of timescales”, Paragraph #5):

> “... We also examine the mechanistic relationship between cycles and memory capacity. We find that randomized null networks that preserve first-level modules and third-level cycle counts reach similar memory capacity to the third-level hierarchical modular reservoirs (Fig. S6). …”

(“Results” section, “Hierarchical modularity supports multitasking”, Paragraph #3):

> “Fig. 5b shows the multitasking score distributions of the three hierarchical levels of modularity across different dynamical regimes. Again, we find that higher-order hierarchical modular networks consistently outperform their lower-order counterparts ($p < 0.05$, $CLES \geq 59.18\%$ for all two-tailed, Wilcoxon-Mann-Whitney two-sample rank-sum tests), including degree-preserving random networks (Fig. S3b; $p < 10^{-9}$, $CLES \geq 75.75\%$, for all two-tailed, Wilcoxon-Mann-Whitney two-sample rank-sum tests) and cycles-preserving random networks (Fig. S6b; $p < 0.01$, $CLES \geq 63.04\%$, for all two-tailed, Wilcoxon-Mann-Whitney two-sample rank-sum tests, except at $\alpha = 1.2$).”

(“Methods” section, “Cycles-preserving null model”):

> “To randomize a third-level hierarchical modular network while preserving first-level modules and cycle counts, we start by applying degree-preserving rewiring with the additional constraint that the number of edges within and between modules is preserved. We then continue to perform degree and modularity-preserving double-edge swaps but additionally condition swap acceptance on a probabilistic Metropolis criterion according to a simulated annealing schedule as in Milisav et al. (2025). Here, we define the cost function as the sum of squared differences in counts for cycles of length 3 and 4.”

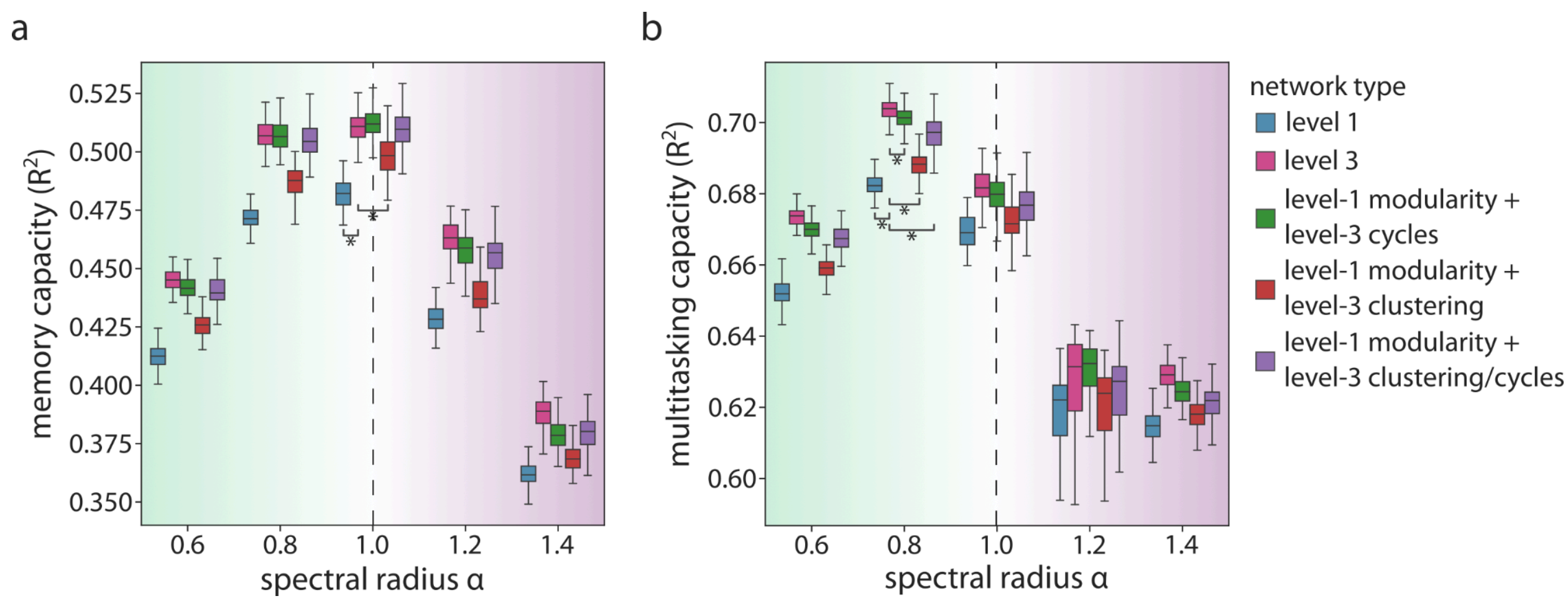

Figure S6 **Modularity-, cycles-, and clustering-preserving null models** | Memory capacity (a) and multitasking score (b) as a function of the *α* parameter for level-1 networks (blue), level-3 networks (magenta) and modularity-, cycles- and/or clustering-preserving randomized surrogates (green, red, and purple). Clustering-preserving null networks were generated using the same approach as for cycles-preserving null networks. Asterisks indicate statistical significance of differences between level-3 networks and other network types according to Wilcoxon-Mann-Whitney tests ($p < 10^{-6}$). Significance is only shown for the highest performing regime.

Milisav, F., Bazinet, V., Betzel, R. F., & Misic, B. (2025). A simulated annealing algorithm for randomizing weighted networks. *Nature Computational Science*, *5*(1), 48-64.

*** Also, the performance difference is statistically significant but effect sizes appear modest (~10–20% gains). For Nature Communications, it must be clarified whether this is functionally meaningful.**

We would like to reiterate that the goal of this paper is not to propose a new state-of-the-art reservoir architecture, but rather to investigate the function of hierarchical modularity. In line with this approach, we demonstrate a clear functional advantage of hierarchical modularity under controlled conditions. We report effect sizes relative to stringent benchmarks, e.g., modularity-preserving nulls, and our results compare favorably to previous work in neuromorphic reservoir computing (Damicelli et al., 2022; Fakhar et al., 2025; Goulas et al., 2021; Loeffler et al., 2021; Rodriguez et al., 2019; Suárez et al., 2021). Following best practices in machine learning, we perform random hyperparameter optimization and statistical significance testing (Ulmer et al., 2022).

While the utility of a hierarchical modular architecture remains to be explored for applied settings, we now extend our results to more complex and realistic chaotic system prediction tasks (see above). The robustness of the computational advantage of hierarchical modularity across numerous modeling scenarios suggests that it could constitute a functionally meaningful neuromorphic inductive bias even for real-world tasks.

Damicelli, F., Hilgetag, C. C., & Goulas, A. (2022). Brain connectivity meets reservoir computing. *PLoS Computational Biology*, *18*(11), e1010639.

Fakhar, K., Akarca, D., Luppi, A. I., Oldham, S., Hadaeghi, F., Vértes, P. E., ... & Astle, D. (2025). Human cortical networks trade communication efficiency for computational reliability. *bioRxiv*, 2025-12.

Goulas, A., Damicelli, F., & Hilgetag, C. C. (2021). Bio-instantiated recurrent neural networks: Integrating neurobiology-based network topology in artificial networks. *Neural Networks*, *142*, 608-618.

Loeffler, A., Zhu, R., Hochstetter, J., Diaz-Alvarez, A., Nakayama, T., Shine, J. M., & Kuncic, Z. (2021). Modularity and multitasking in neuro-memristive reservoir networks. *Neuromorphic Computing and Engineering*, *1*(1), 014003.

Rodriguez, N., Izquierdo, E., & Ahn, Y. Y. (2019). Optimal modularity and memory capacity of neural reservoirs. *Network Neuroscience*, *3*(2), 551-566.

Suárez, L. E., Richards, B. A., Lajoie, G., & Misic, B. (2021). Learning function from structure in neuromorphic networks. *Nature Machine Intelligence*, *3*(9), 771-786.

Ulmer, D., Hardmeier, C., & Frellsen, J. (2022). Deep-significance-easy and meaningful statistical significance testing in the age of neural networks. *arXiv preprint arXiv:2204.06815*.

*** Signed/negative weights are only addressed briefly in Supplementary Fig. S8, where the effect of hierarchy largely disappears. This raises a major limitation: brain networks are inhibitory–excitatory, and many learning systems require inhibitory edges. The main conclusions of the paper may not be valid when we also consider negative weights.**

We agree with the Reviewer that the absence of memory improvement for hierarchical modularity in fully excitatory-inhibitory networks is a notable limitation of its functional

advantages. However, we would like to point out that brain networks show structured inhibition, that is, inhibitory connections are overwhelmingly local. Here, we consider hierarchical modularity as a macroscale organizational principle of the connectome. Treating long-range interregional connections as exclusively excitatory is a ubiquitous modeling assumption across a wide variety of connectome-based dynamic models (Breakspear et al., 2003; Chaudhuri et al., 2015; Kringelbach et al., 2023; Jansen & Rit, 1995; Pope et al., 2021; Wilson & Cowan, 1972, 1973; Wong & Wang, 2006). Notably, when we allow negative weights only in low-level modules, the memory benefits of hierarchical modularity are recovered. This indicates that the functional advantage of hierarchical modularity is limited to structured, biomimetic inhibition patterns. Nevertheless, we emphasize this limitation in both the *Results* and *Discussion* sections:

("Results" section, "Sensitivity analyses", Paragraph #1):

> "… However, no significant difference was found between levels of the modularity hierarchy when including negative weights ($\boldsymbol{W} \sim U(-1, 1)$). Furthermore, asymmetric networks including negative weights can achieve near perfect performance at high $\boldsymbol{\alpha}$ values. Importantly, note that the goal of this paper is not to present hierarchical modularity as a new state-of-the-art reservoir architecture, but to study its function in various modeling frameworks. In line with this objective, we take a more biomimetic approach to negative weight placement, constraining them to local within-module connections. Interestingly, this approach restores the effect of hierarchical modularity, despite a drop in performance compared to allowing negative weights throughout the network. Altogether, these results confirm that the computational advantages of hierarchical modularity are robust to numerous modeling settings."

("Discussion" section, Paragraph #8):

> "... Second, we find that the effect of hierarchical modularity on memory capacity only generalizes to signed networks when negative weights are introduced in first-level modules. This indicates that random placement of negative weights might effectively blur a binary hierarchical modular organization. Given the importance of negative connections for representation capacity (Wang et al., 2023), future work should explore how to most efficiently integrate them in hierarchical modular architectures. Interestingly, placing negative weights only in low-level modules reflects the prevalent modeling assumption that the brain is organized in local populations of interacting excitatory and inhibitory neurons, coupled exclusively through excitatory

> long-range connections (Wilson & Cowan, 1972, 1973). More broadly, these results pave the way for more complex multi-region "network of networks" RNN models (Perich & Rajan, 2020)."

Breakspear, M., Terry, J. R., & Friston, K. J. (2003). Modulation of excitatory synaptic coupling facilitates synchronization and complex dynamics in a biophysical model of neuronal dynamics. *Network: Computation in Neural Systems*, *14*(4), 703.

Chaudhuri, R., Knoblauch, K., Gariel, M. A., Kennedy, H., & Wang, X. J. (2015). A large-scale circuit mechanism for hierarchical dynamical processing in the primate cortex. *Neuron*, *88*(2), 419-431.

Jansen, B. H., & Rit, V. G. (1995). Electroencephalogram and visual evoked potential generation in a mathematical model of coupled cortical columns. *Biological cybernetics*, *73*(4), 357-366.

Kringelbach, M. L., Perl, Y. S., Tagliazucchi, E., & Deco, G. (2023). Toward naturalistic neuroscience: Mechanisms underlying the flattening of brain hierarchy in movie-watching compared to rest and task. *Science advances*, *9*(2), eade6049.

Perich, M. G., & Rajan, K. (2020). Rethinking brain-wide interactions through multi-region 'network of networks' models. *Current opinion in neurobiology*, *65*, 146-151.

Pope, M., Fukushima, M., Betzel, R. F., & Sporns, O. (2021). Modular origins of high-amplitude cofluctuations in fine-scale functional connectivity dynamics. *Proceedings of the National Academy of Sciences*, *118*(46), e2109380118.

Wang, Q., Powell, M. A., Geisa, A., Bridgeford, E., Priebe, C. E., & Vogelstein, J. T. (2023). Why do networks have inhibitory/negative connections?. In *Proceedings of the IEEE/CVF International Conference on Computer Vision* (pp. 22551-22559).

Wilson, H. R., & Cowan, J. D. (1972). Excitatory and inhibitory interactions in localized populations of model neurons. *Biophysical journal*, *12*(1), 1-24.

Wilson, H. R., & Cowan, J. D. (1973). A mathematical theory of the functional dynamics of cortical and thalamic nervous tissue. *Kybernetik*, *13*(2), 55-80.

Wong, K. F., & Wang, X. J. (2006). A recurrent network mechanism of time integration in perceptual decisions. *Journal of Neuroscience*, *26*(4), 1314-1328.

*** Fig 3 – Timescales:**
a) The claim that hierarchical networks yield a “pool of timescales” is based on variability in autocorrelation decay constants. But variability alone does not prove functional benefit unless linked to task performance.

We agree with the Reviewer. This is why we explicitly relate memory capacity to timescale variability in figure S5a. We find a significant positive relationship between the two (Speaman’s rho = 0.52, $p < 10^{-21}$).

(“Results” section, “Hierarchical modularity generates a pool of timescales”, Paragraph #5):

> “Overall, these results point to recurrence and temporal expansion as potential substrates of memory capacity in hierarchical modular networks. To validate this hypothesis, we relate timescale variability and motif composition to memory capacity across all reservoirs (Fig. S5). We consistently find that features related to hierarchical modular reservoirs also relate to enhanced memory capacity.”

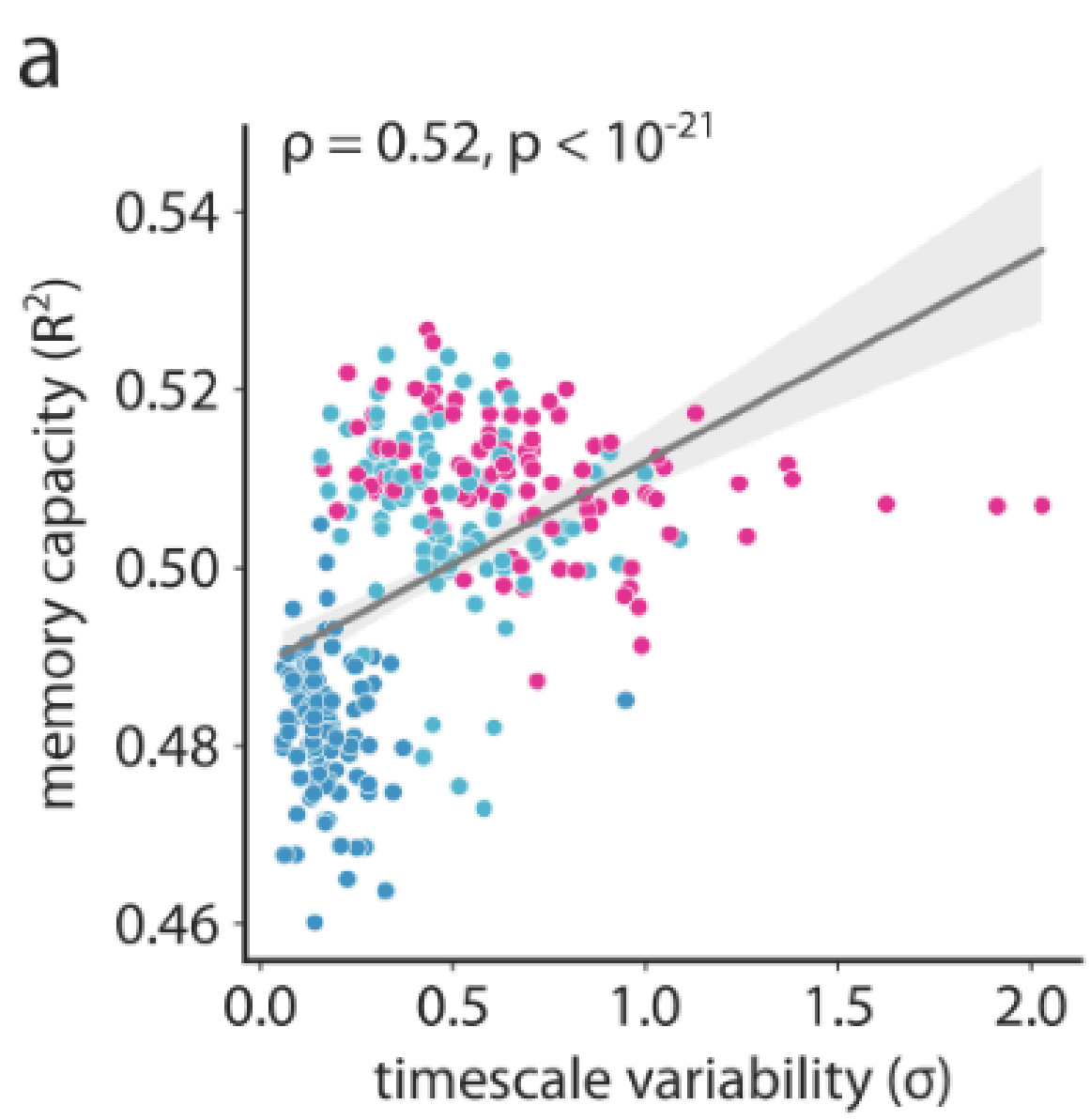

b) The timescale measure is highly sensitive to noise and spectral radius scaling. No robustness checks with alternative definitions (e.g., exponential fit vs spectral entropy, power-law fits) are presented.

Note that timescales are measured at α=1 across all hierarchical levels. The spectral radius is therefore fixed. Furthermore, we use the same training and testing signals

across all hierarchical levels and no noise is injected in either signal. The signals only differ across networks within each level's ensemble.

Nevertheless, we replicate our results using spectral entropy as per the Reviewer's recommendation. For each output node time series, we estimate power spectral density (PSD) using Welch's method and compute normalized spectral entropy. We find strong negative relationships between timescales derived from exponential fits of the autocorrelation function (ACF) and spectral entropy, for both the synthetic graphs (mean Spearman's rho = -0.90, standard deviation = 0.12) and the empirical connectome (Spearman's rho = -0.88, $p < 10^{-132}$; see scatter plot on the left). This is in line with a neural-like Lorentzian PSD and an exponentially decaying ACF (Gao et al., 2020). We also replicate the increase in variability with additional levels of the modularity hierarchy for spectral entropy (see distributions on the right; $p < 10^{-6}$, CLES = 70.29-99.98% for all two-tailed, Wilcoxon-Mann-Whitney two-sample rank-sum tests).

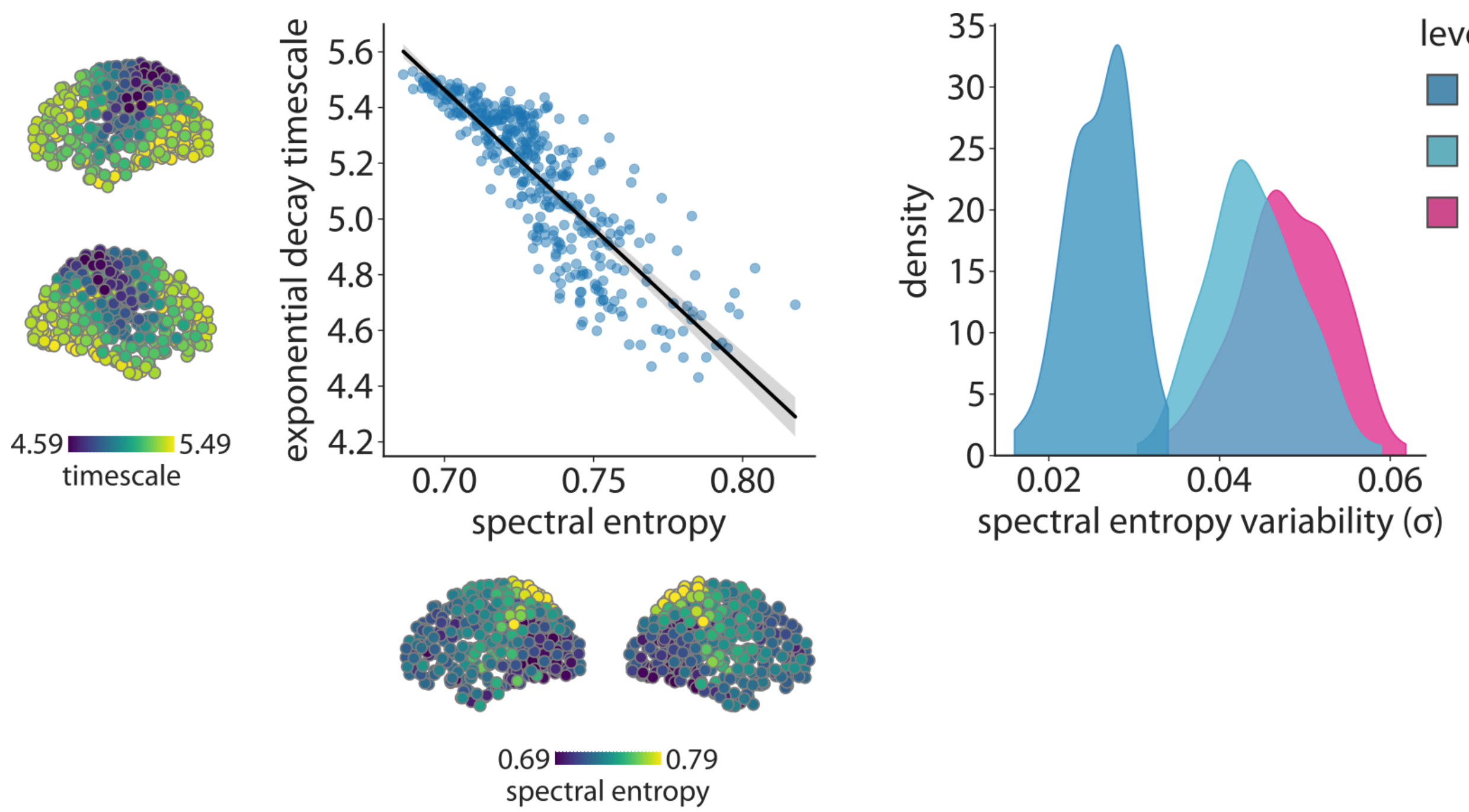

We hope that these results reassure the Reviewer of the robustness of our findings. Note however that we do not include them in the manuscript as spectral entropy is not a direct measure of timescale.

Gao, R., Van den Brink, R. L., Pfeffer, T., & Voytek, B. (2020). Neuronal timescales are functionally dynamic and shaped by cortical microarchitecture. *elife*, *9*, e61277.

*** Fig. 4: Motif enrichment is reported but not normalized against randomized graphs that preserve motif distributions at lower levels. Without motif-specific nulls, the interpretation that "reciprocal/cyclic motifs drive memory" is speculative.**

The lower-level strictly modular networks already act as randomized graphs that preserve motif distributions at lower levels. Lower-level modularity is preserved across all levels of the hierarchy and we compare the resulting motif compositions. That is, our reports of motif enrichment at level 3 are already relative to motif enrichment at level 1 via statistical significance testing.

Note that we also find significant relationships between motif counts and memory capacity. These correlations are provided in figure S5 together with correlations between timescale variability and memory capacity (figure S5a; see above).

The link between cycles and information storage is cited but no direct task-based manipulation (e.g., remove cycles of length 3–5 and retest memory capacity) is attempted.

We thank the Reviewer for this suggestion. To mechanistically evaluate the effect of cycles on memory capacity in hierarchical modular reservoirs, we develop a perturbation experiment that directly removes cycles of length 3-5. Briefly, we use constrained rewiring, only accepting double-edge swaps if they do not create new cycles. We only test cycles of length 3, as extending the constraint to longer lengths is too restrictive, i.e., valid swaps are rapidly exhausted, preventing meaningful perturbation. Nevertheless, we observe that cycles of lengths 4 and 5 are concomitantly destroyed up to a certain number of swaps. Therefore, we logarithmically sample snapshots along the rewiring trajectory up to the point at which the average number of cycles of any length between 3 and 5 starts increasing. This yields 7 ensembles of perturbed hierarchical modular networks with monotonically decaying cycle counts. We find that the destruction of cycles is systematically accompanied by a drop in memory capacity, reinforcing their importance for information storage in hierarchical modular reservoirs. Below, we detail the addition of these findings to the manuscript:

("Results" section, "Hierarchical modularity generates a pool of timescales", Paragraph #5):

> "... We also examine the mechanistic relationship between cycles and memory capacity. We find that randomized null networks that preserve first-level modules and third-level cycle counts reach similar memory capacity

to the third-level hierarchical modular reservoirs (Fig. S6a). Furthermore, we find that destroying cycles via degree-preserving rewiring systematically reduces the memory capacity of hierarchical modular reservoirs across all dynamical regimes (Fig. S7). Altogether, these results suggest a causal role of cycles in hierarchical modular reservoir memory capacity."

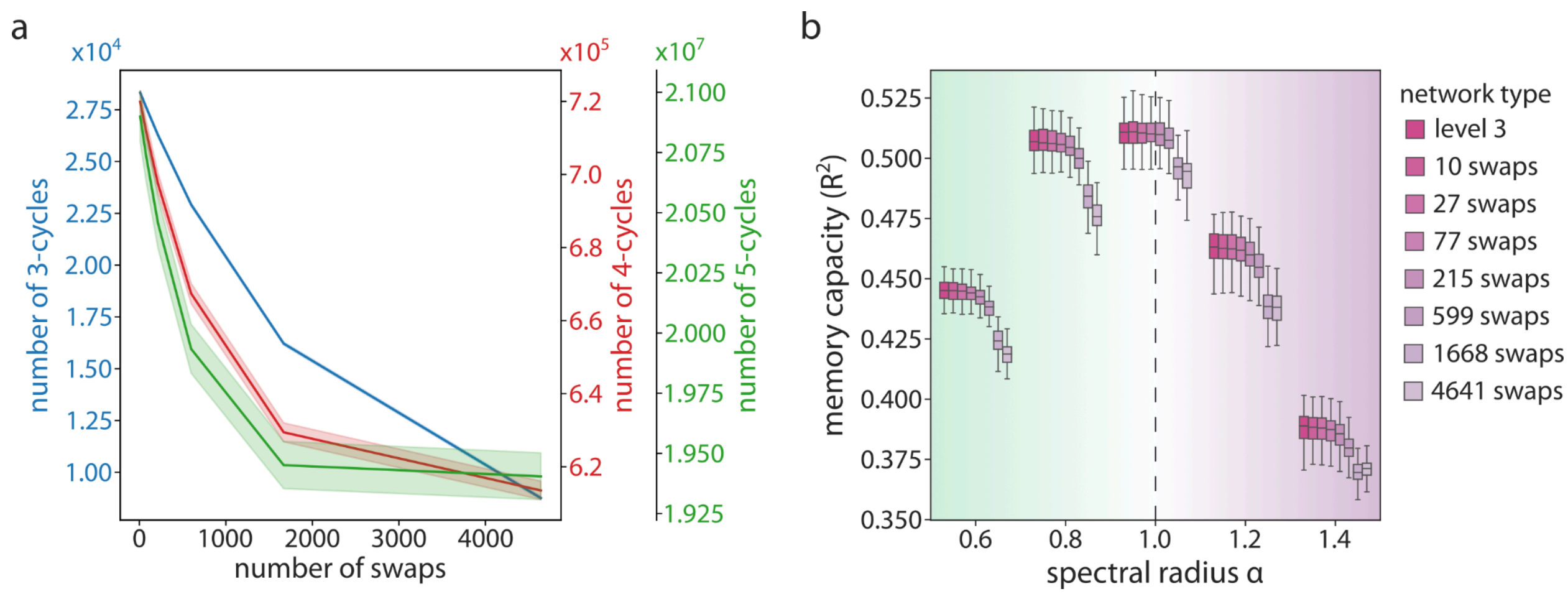

Figure S7 **Cycles perturbation experiment |** To remove cycles of length 3-5, we use degree-preserving rewiring with the additional constraint that the double-edge swaps do not create new cycles of length 3. We limit the constraint to 3-cycles as extending it to longer lengths rapidly exhausts valid swaps, preventing meaningful perturbation. Nevertheless, we observe that cycles of lengths 4 and 5 are initially concomitantly destroyed. We stop the procedure when the average number of cycles of any length between 3 and 5 starts increasing. (a) Number of 3- (blue), 4- (red), and 5-cycles (green) as a function of the number of constrained swaps. (b) Memory capacity (R^2) as a function of the α parameter for level-3 networks and their rewired surrogates.

*** Fig. 5: The multitasking framework is adapted from Loeffler et al. 2021. However, only one multitask configuration is shown prominently; others are relegated to Supplementary (S4, S5). The key issue is whether hierarchy provides robust advantage across all multitask scenarios. In Fig. S5 (8 tasks), third-level hierarchy gives no extra improvement over two levels, raising doubt that "deeper hierarchy" adds real benefit.**

Note that the second level of hierarchy systematically improves performance over strictly modular networks. The computational advantage of hierarchical modularity is therefore robust across multitasking scenarios. The results simply indicate that the optimal number of levels might be task-specific.

However, we agree with the Reviewer that our results also show that additional hierarchical levels are not always beneficial, which is why we report that:

“Importantly, since performance rapidly saturates with additional hierarchical levels, our findings also suggest that a deep fractal organization might not be necessary to achieve performance improvements.”

*** Fig 6: The hierarchical null model is a methodological contribution. However, it is unclear if it preserves other important structural statistics (edge length distribution, degree assortativity). If not, the observed differences may reflect confounds.**

To the contrary, we believe that explicitly preserving additional network features would introduce confounds in the null networks. We propose the hierarchical modular null model to test if the computational benefits of hierarchical modularity extend to empirical heterogeneous connectivity patterns. To answer this question, we design null models that embody a hierarchy of constraints, i.e. modularity and hierarchical modularity. This allows us to disentangle the specific contribution of an additional level of nested modules over the first level alone, similar to our analysis in stochastic block models, but extended to empirical heterogeneous modules. Adding additional constraints is not only difficult in implementation, but would additionally overload the null hypothesis, preventing us from specifically allocating which part of the effect can be attributed to hierarchical modularity as opposed to the other preserved features. Building additional incremental null models that incorporate other features one at a time could address this problem, but we believe it to be outside the scope of this paper, which focuses on hierarchical modularity.

Nevertheless, for clarification, we compare the edge length distributions and degree assortativity of the empirical and the null networks. We find shorter edge lengths (see cumulative distribution functions on the left) and lower assortativity (see assortativity distributions on the right) in the empirical connectome, indicating that these features are not preserved. The brain’s shorter edge lengths speak to the economical trade-off it must compose with (Bullmore & Sporns, 2012), which might partially explain its lower memory capacity. We expand on this perspective in our response to the Reviewer’s following comment.

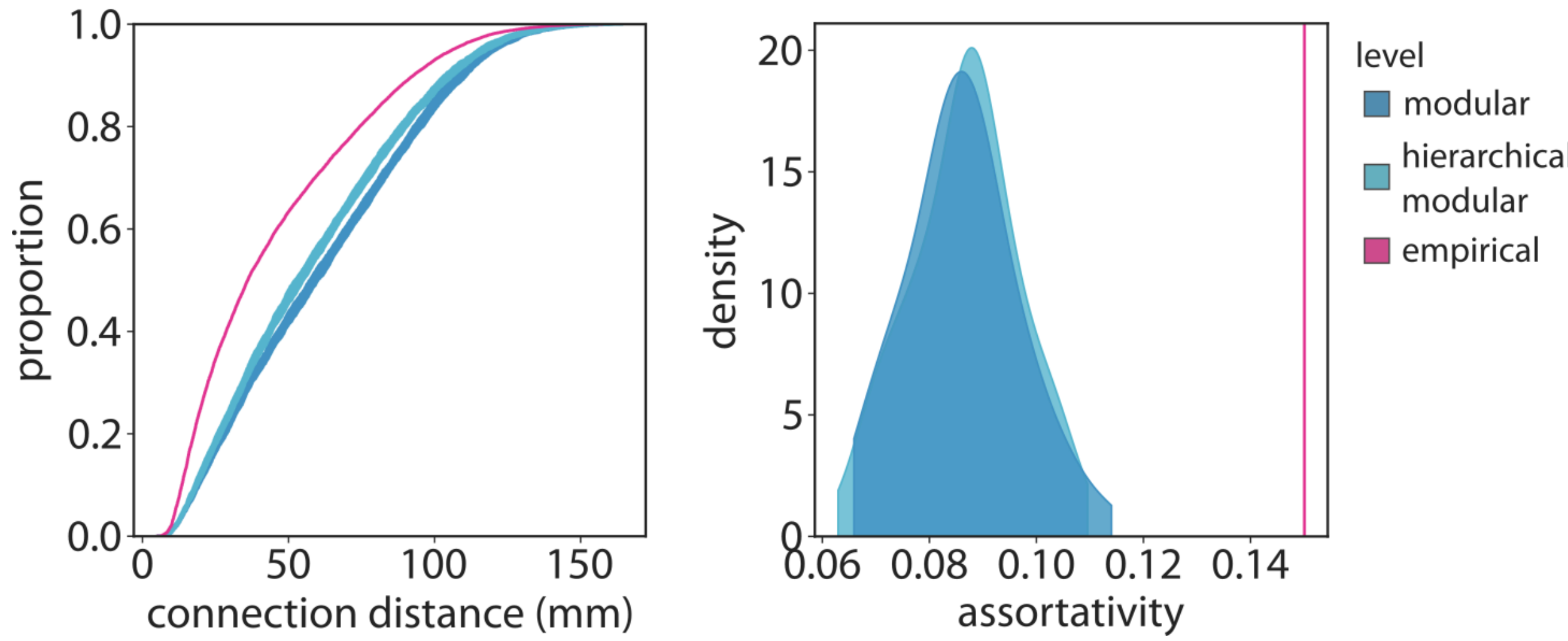

Bullmore, E., & Sporns, O. (2012). The economy of brain network organization. *Nature reviews neuroscience*, *13*(5), 336-349.

*** The result that null networks outperform empirical connectomes in stable regimes (α < 1) is brushed aside as “other topological features are detrimental.” This undermines the biomimetic argument: why should the real brain underperform synthetic surrogates if hierarchical modularity is its key advantage?**

We agree with the Reviewer that our interpretation of this result can be elaborated. We believe that some topological features of the empirical connectome might be detrimental to memory capacity because the brain’s connectivity must navigate multiple trade-offs. The most established is unequivocally the economical trade-off, which dictates that the optimization of advantageous network properties be negotiated with an imperative towards minimization of wiring costs (Bullmore & Sporns, 2012). In line with this principle, we normalize the memory capacity of connectome-informed reservoirs by their average connection length (Suárez et al., 2021). This approach reveals that the normalized memory capacity of the empirical connectome is considerably superior to that of (hierarchical) modularity-preserving nulls (see below), reinforcing the idea that the brain balances computational performance with wiring cost. More recent reports suggest that other trade-offs could also be at play, with the brain potentially balancing computational performance and reliability (Fakhar et al., 2025; Kawai et al., 2019; Liu et al., 2026). In the revised manuscript, we now elaborate our interpretation to suggest trade-off effects and report these new findings:

("Results" section, "Hierarchical modularity in the human connectome", Paragraph #4):

"... In particular, this result might reflect the numerous trade-offs that brain wiring must navigate to balance computational performance with robustness (Fakhar et al., 2025; Kawai et al., 2019; Liu et al., 2026) and wiring costs (Bullmore & Sporns, 2012). To account for the metabolic and material costs of longer connections, we normalize memory capacity by the average Euclidean length of reservoir edges, following Suárez et al. (2021). We find that the empirical connectome significantly outperforms the modular and hierarchical modular null networks across α values when controlling for wiring costs (Fig. S10; $p < 10^{-30}$, $CLES \geq 97.27\%$ for all two-tailed, Wilcoxon-Mann-Whitney two-sample rank-sum tests), providing a better economical trade-off."

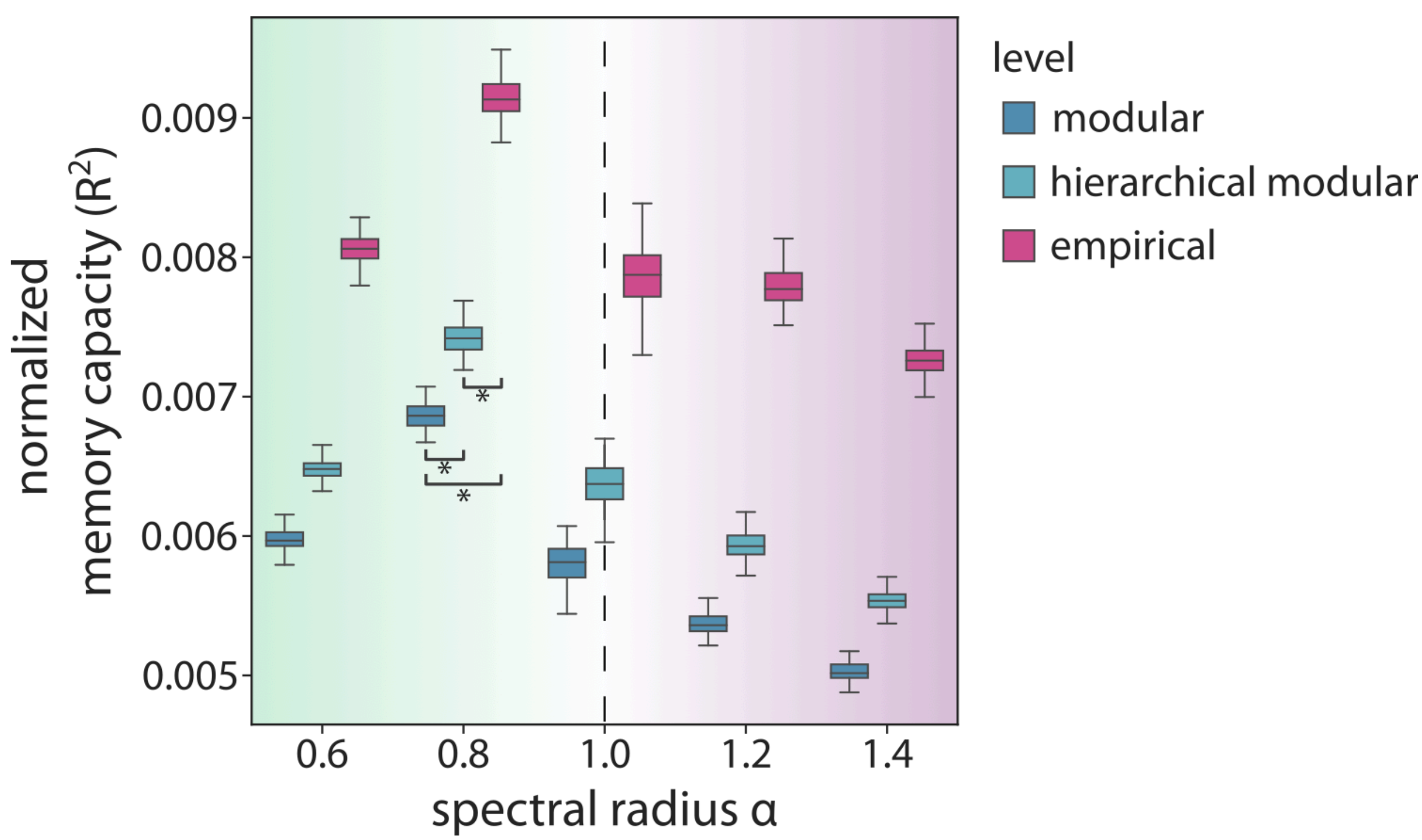

Figure S10 **Trade-off between memory capacity and wiring cost in the human connectome** | Memory capacity (R^2) normalized by average Euclidean edge length as a function of the α parameter across hierarchical levels. Asterisks indicate statistical significance of differences between levels according to Wilcoxon-Mann-Whitney tests ($p < 10^{-33}$). Significance is only shown for the highest performing regime.

Note also that the empirical connectome is not necessarily "more hierarchical modular" than the hierarchical modularity-preserving null. The null is meant to capture the full empirical modularity hierarchy to the extent allowed by the Louvain algorithm.

Bullmore, E., & Sporns, O. (2012). The economy of brain network organization. *Nature reviews neuroscience*, *13*(5), 336-349.

Fakhar, K., Akarca, D., Luppi, A. I., Oldham, S., Hadaeghi, F., Vértes, P. E., ... & Astle, D. (2025). Human cortical networks trade communication efficiency for computational reliability. *bioRxiv*, 2025-12.

Kawai, Y., Park, J., & Asada, M. (2019). A small-world topology enhances the echo state property and signal propagation in reservoir computing. *Neural Networks*, *112*, 15-23.

Liu, Y., Wei, Y., & Peng, H. (2026). From local motifs to global dynamical stability in the mouse brain connectome. *bioRxiv*, 2026-02.

Suárez, L. E., Richards, B. A., Lajoie, G., & Misic, B. (2021). Learning function from structure in neuromorphic networks. *Nature Machine Intelligence*, *3*(9), 771-786.

*** Timescale comparison to MEG data shows a correlation ρ ≈ 0.44, which is statistically strong but moderate. The null distributions (Fig. 6d right) show that hierarchical nulls achieve similar correlations, so hierarchy alone cannot explain empirical timescales.**

It is important to note that we never interpret hierarchy as a standalone feature. We study hierarchical modularity, which cannot be isolated from modularity since we operationalize it as a hierarchy of nested modules. Therefore, we do not claim that hierarchy alone can explain empirical timescales. We also do not claim that hierarchical modularity alone shapes empirical timescales, notably because the relationship between synthetic and empirical timescales is moderate even for empirical connectivity patterns, as pointed out by the Reviewer. Other features, such as spatial embedding, might additionally play a role (Shi et al., 2023). Yet, we suggest that "hierarchical modularity could contribute to shaping gradients of intrinsic neural timescales in the brain" precisely because hierarchical modularity-preserving null networks achieve similar correlations as those observed for the empirical connectome. That is, we cannot reject the null hypothesis that the relationship between synthetic and empirical timescales is due to hierarchical modularity ($p_{rewired} \approx 0.13$).

Shi, Y. L., Zeraati, R., Levina, A., & Engel, T. A. (2023). Spatial and temporal correlations in neural networks with structured connectivity. *Physical review research*, *5*(1), 013005.

Overall assessment:
In my opinion the paper offers a carefully executed demonstration that hierarchical modularity improves reservoir comp performance, but its big picture contribution is less significant than presented. The novelty lies in formalizing hierarchy within SBM reservoirs and introducing nulls, rather than in the central idea that hierarchy supports memory and multitasking. The work is solid, but whether it rises to the level of Nature Communications depends on how the editors weigh methodological rigor against conceptual novelty. Without clearer differentiation from prior hierarchical RC work and without more challenging demonstrations, the contribution risks being seen as incremental.

We thank the Reviewer for acknowledging the methodological rigor of our work. We hope that our clarifications have convinced the Reviewer that hierarchical modularity is fundamentally different from hierarchy as it was operationalized in previous reservoir computing studies. In the revised manuscript, we now also demonstrate that the computational advantages of hierarchical modularity can extend to more complex time series prediction tasks.

Reviewer #2 (Remarks to the Author):

This manuscript reports results of a new mathematical model that investigates the effects of hierarchically organised modular networks on learning and memory capacity, studied using reservoir computing (RC). While all the results are based on simulation, the authors have integrated empirical human connectome data from MEG. The results are original and noteworthy and presented well overall. Below I provide some comments and suggested edits that I hope will serve to improve the manuscript.

We thank the Reviewer for their positive comments and constructive suggestions.

- Methods: Details about the sizes of the modular networks (including the number of output nodes used in the RC tasks) should be mentioned upfront. Some details are only mentioned in sensitivity analysis and in Fig. S7. The number of nodes & edges is important for reproducibility and benchmarking, and also since it determines RC performance.

In the revised manuscript, we now explicitly report network size at the beginning of the *Results*. We also report the number of edges following pruning and the number of output nodes for each task in the *Methods*:

("Results" section, "Generating hierarchical modular reservoirs", Paragraph #2):

> "Specifically, we model three hierarchical levels of modularity. At the first level, we specify 8 densely connected modules of 50 nodes, for a total network size of 400 nodes."

("Methods" section, "Hierarchical modular network model", last paragraph):

> "We implement all stochastic block models in the hierarchy using the openly available NetworkX package (https://networkx.org/documentation/stable/index.html; Hagberg et al., 2008). They all share an expected degree of 62 and an expected density of 15.54%, for a total of 24000 edges. However, note that expected density drops to 9.95%, for a total of approximately 15873 edges following pruning in the main analyses (see *Hyperparameter tuning*)."

(“Methods” section, “Memory capacity”, Paragraph #3):

> “Output nodes correspond to the nodes of all the other 7 50-node modules. A different readout module is trained for each one and final performance is averaged across all readout modules.”

(“Methods” section, “Multitasking”, Paragraph #2):

> “In the 2-task scenario, input nodes for each task correspond to all the nodes in a randomly selected first-level module. The other 3 first-level 50-node modules in the same third-level module are used as output. As for the memory capacity task, a different readout module is trained for each one and final performance is averaged across all readout modules. In the 4-task scenario, input nodes for each task also correspond to all the nodes in a first-level module. The other first-level 50-node module in the same second-level module is used as output. Finally, in the 8-task scenario, an input node is randomly selected in each of the 8 first-level modules. All of the other 49 nodes in each module are used as output.”

- Figs. 2b, 5b & 6c (and supp figs): I suggest removing the title "criticality" since it may be misinterpreted and is anyway not needed in the figures. As the authors state themselves, dynamics are sometimes referred to as critical at alpha=1, but the descriptor criticality could easily be misinterpreted by some neuroscience readers as avalanche criticality, which would be incorrect as no activity propagation is shown. Edge of stability or edge of chaos criticality is OK to make this distinction - see e.g. Kanders et al. "Avalanche and edge-of-chaos criticality do not necessarily co-occur in neural networks." Chaos: An Interdisciplinary Journal of Nonlinear Science 27.4 (2017); and Hochstetter et al. "Avalanches and edge-of-chaos learning in neuromorphic nanowire networks." Nature Communications. 12, 4008 (2021).

In the revised manuscript, we now remove the term “criticality” from the figures and from results descriptions, replacing it with “edge-of-chaos”, as per the Reviewer’s suggestion.

- Fig. 2b & 6c: MC results are shown as R^2, but it would be helpful to show the raw MC values, since this is what is usually reported in RC studies and would make comparisons & benchmarking much easier. These could be supplementary figures. Note that the MC task was originally introduced to evaluate fading memory due to recurrence in RNNs, so a note should be made in relation to Fig. 4 that shows the enhanced recurrent motifs and number of cycles in higher levels as corroborating/explaining Fig.2b.

In figure S2, we now show MC values derived from summation across time lags of squared Pearson correlation coefficients between predicted and target signals.

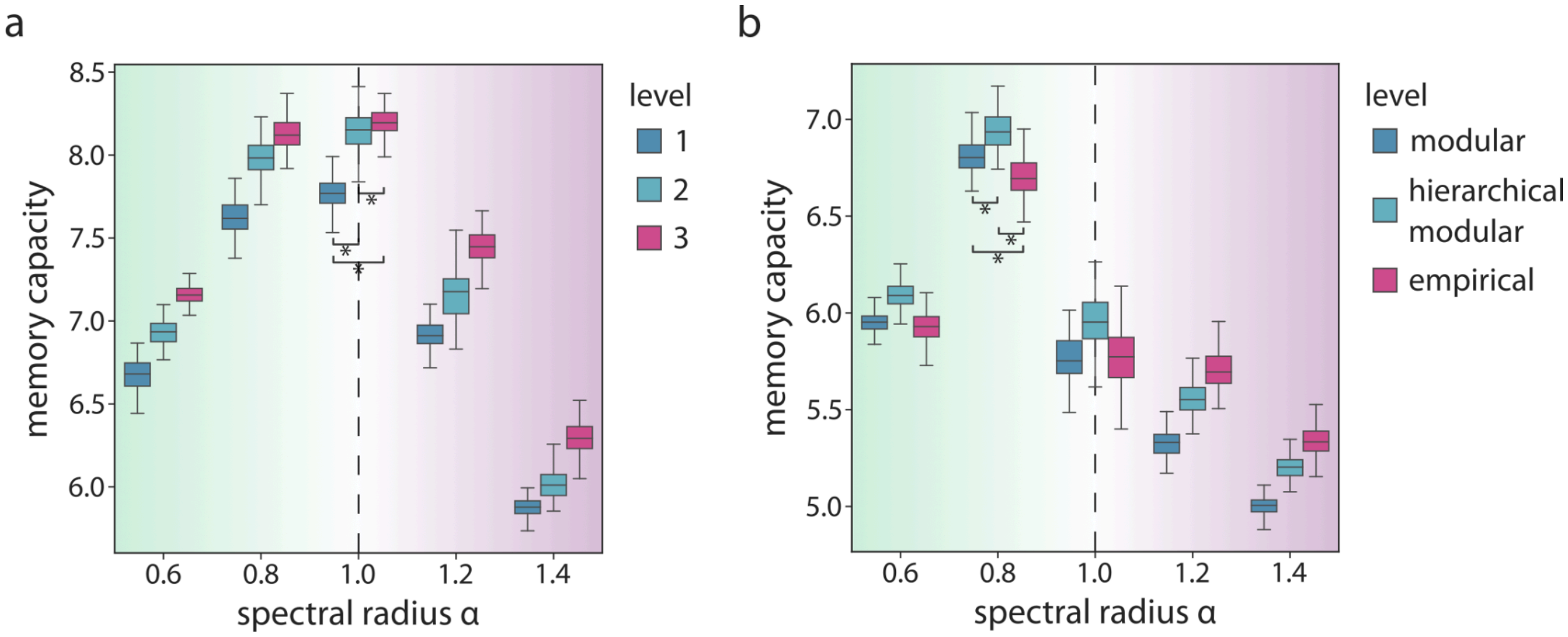

Figure S2 **Standard memory capacity** | (a) Same as in Fig. 2b, but with memory capacity measured as the sum of squared Pearson correlation coefficients across all time delays. (b) Same as in Fig. 6c, but with memory capacity measured as the sum of squared Pearson correlation coefficients across all time delays. Asterisks indicate statistical significance of differences between levels according to Wilcoxon-Mann-Whitney tests ($p < 0.01$). Significance is only shown for the highest performing regime.

We also share the Reviewer's hypothesis of a relationship between recurrent motifs and memory capacity. This is why we explicitly relate them in figure S5. Furthermore, in response to comments from Reviewer #1, we now additionally probe this relationship mechanistically, using cycles-preserving null models and direct perturbation experiments (see above).

- Fig. 3b: can you please also provide 2 additional supplementary figures showing corresponding output node states for alpha<1 and alpha >1.

In figure S4, we now show output node states for alpha = 0.8 and alpha = 1.2:

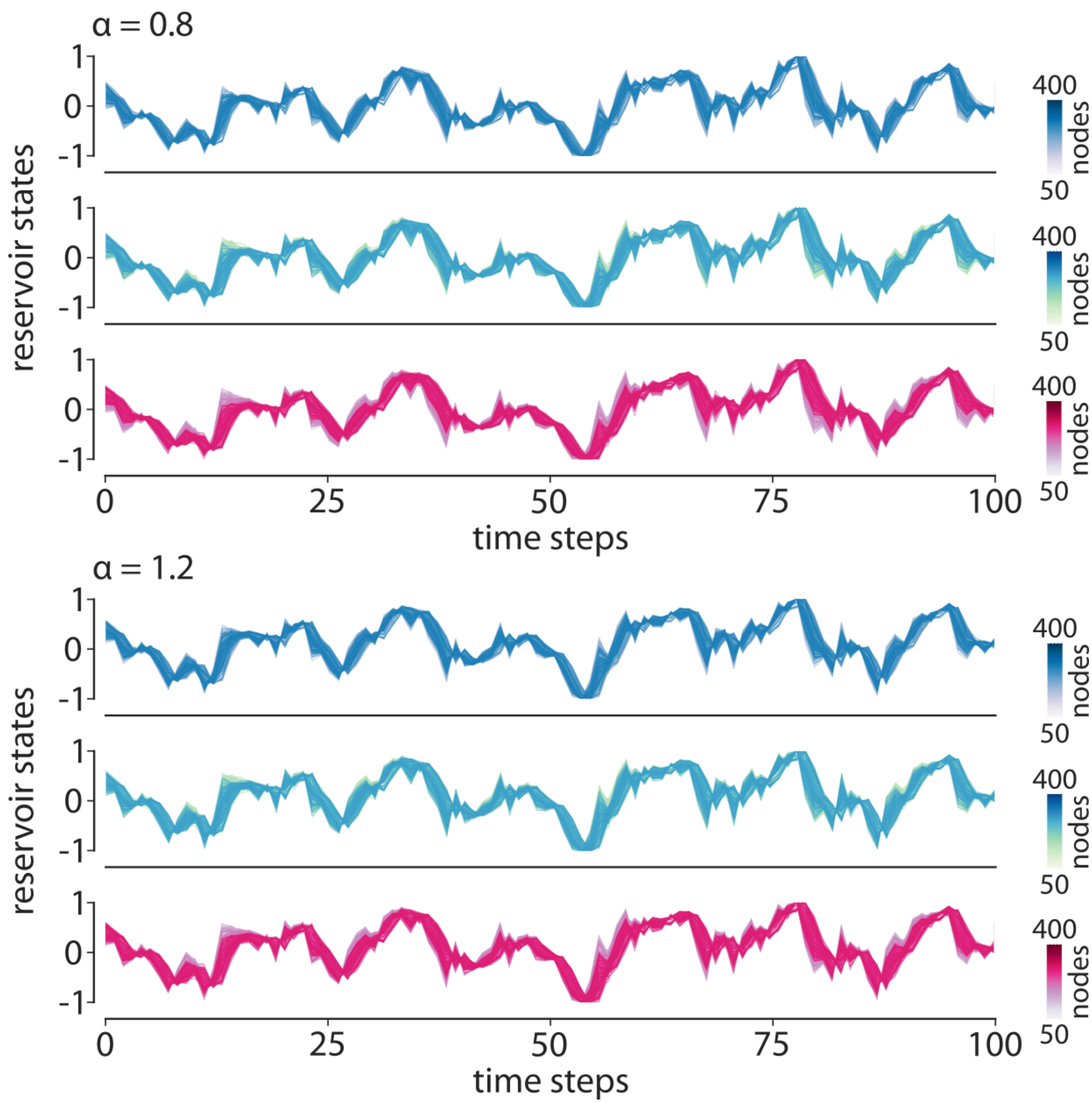

Figure S4 **Example reservoir time series** | Output node states at α = 0.8 (a) and α = 1.2 (b) for an example reservoir of each of the three levels. The time series were rescaled between -1 and 1 to emphasize differences. Different colours correspond to different nodes.

- Fig. 4: can you add a legend at the bottom rigfht and also assuming this is for alpha=1 networks, add that to the caption.

The reported network features do not take into account edge weights, making them valid for all alpha values. Indeed, scaling the spectral radius does not affect network topology. While we do not report the spectral radius for this reason, we have now added

a legend mapping each colour to the corresponding level of hierarchy, as per the Reviewer's recommendation:

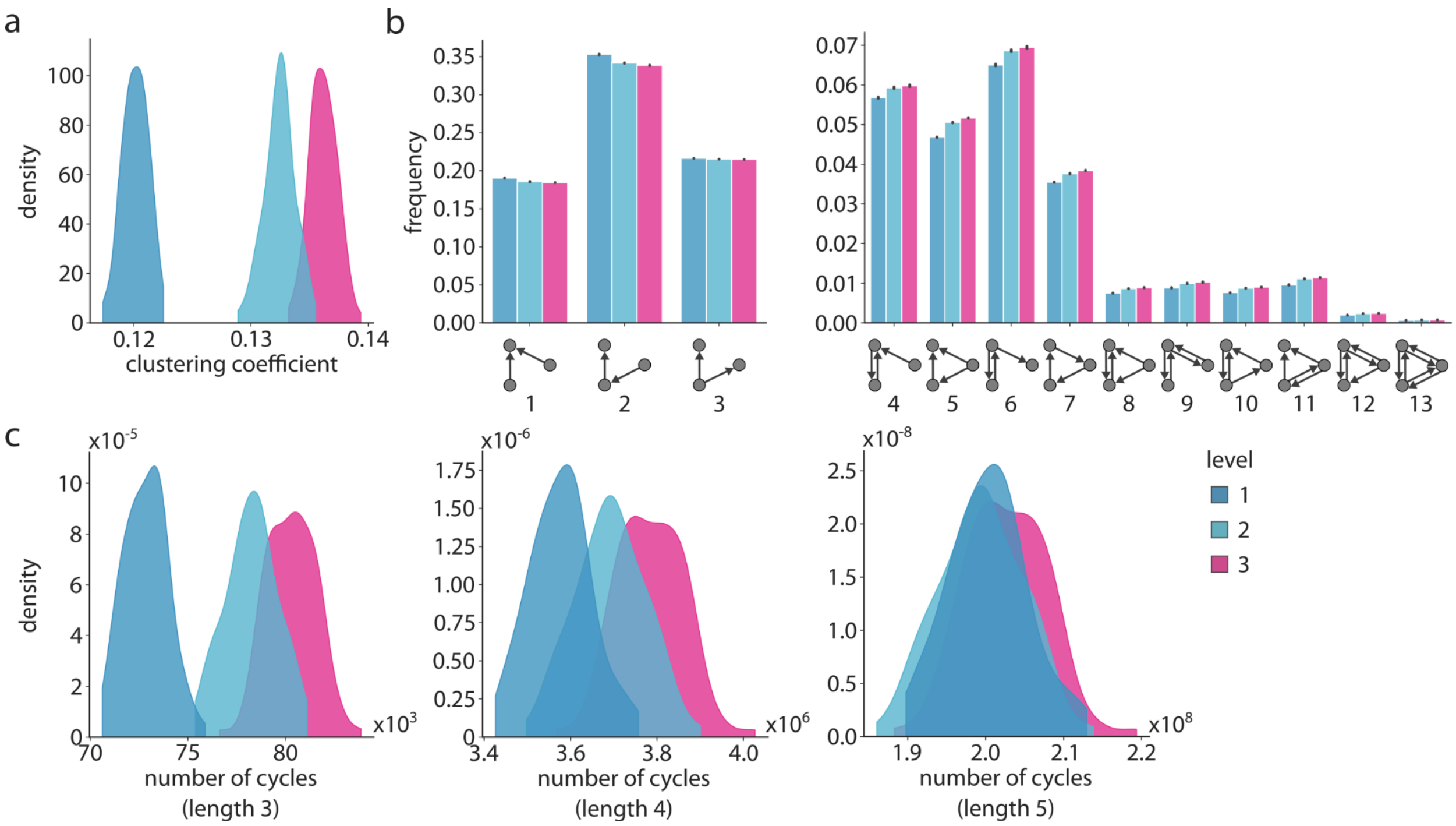

- Multitasking results: these are very interesting. Are you able to provide further explanation for why the more complex multitasking scenarios in Figs. S4 & S5 show performance peaking at alpha=1, whereas Fig. 5b shows the peak at alpha=0.8? Also, did you try to regress to a different waveform to check whether eoc criticality only benefits the more computationally complex task of regressing to a square wave (as in Hochstetter et al.)? Note that the two multitasking tasks in Loeffler et al. were selected because they operate on different dynamical timescales: MC is fast, whereas waveform regression is relatively slow, and they leverage different resources - recurrent loops vs. internal dynamics. Here, hierarchical modularity seems to contribute to both. This might be worth mentioning if the authors find it interesting.

We thank the Reviewer for this comment as it allowed us to identify a bug in our code. We inadvertently implemented a one-step-ahead prediction task instead of the waveform regression task. That is, the task consisted in a slight phase shift conversion of the sinusoidal input instead of a regression to a square signal. All figures and results have been corrected in the revised manuscript (see below). Importantly, the functional advantage of hierarchical modularity is preserved, indicating robustness to the complexity of the non-linear transformation task.

Interestingly, peak performance is now observed in the stable alpha=0.8 regime not only for the main 2-task multitasking experiment, but also in the 8-task scenarios. Unfortunately, we were not able to identify the principle underlying these changes in optimal dynamical regime. Values of 0.8 and 1 were both identified as optimal across task complexity levels including nonlinear transformation complexity (phase shift conversion vs more complex waveform regression), number of tasks, and task category interleaving. Differences in optimal dynamical regime therefore seem to depend on more minute task idiosyncrasies.

We also thank the Reviewer for pointing out the difference in timescales between the two tasks. As noted by the Reviewer in the next comment, our tanh units do not have internal memory capability like Loeffler's memristive junctions. The nonlinear transformation task therefore does not leverage that resource. Nevertheless, the fact that hierarchical modular reservoirs might improve temporal processing across multiple task-specific timescales is noteworthy in of itself and we now emphasize this aspect of the task when introducing it:

("Results" section, "Hierarchical modularity supports multitasking", Paragraph #1):

> "... Furthermore, the two tasks operate on different timescales: the memory capacity task requires the reservoir to store fast random fluctuations, whereas the non-linear transformation task has slower dynamics (Loeffler et al., 2021)"

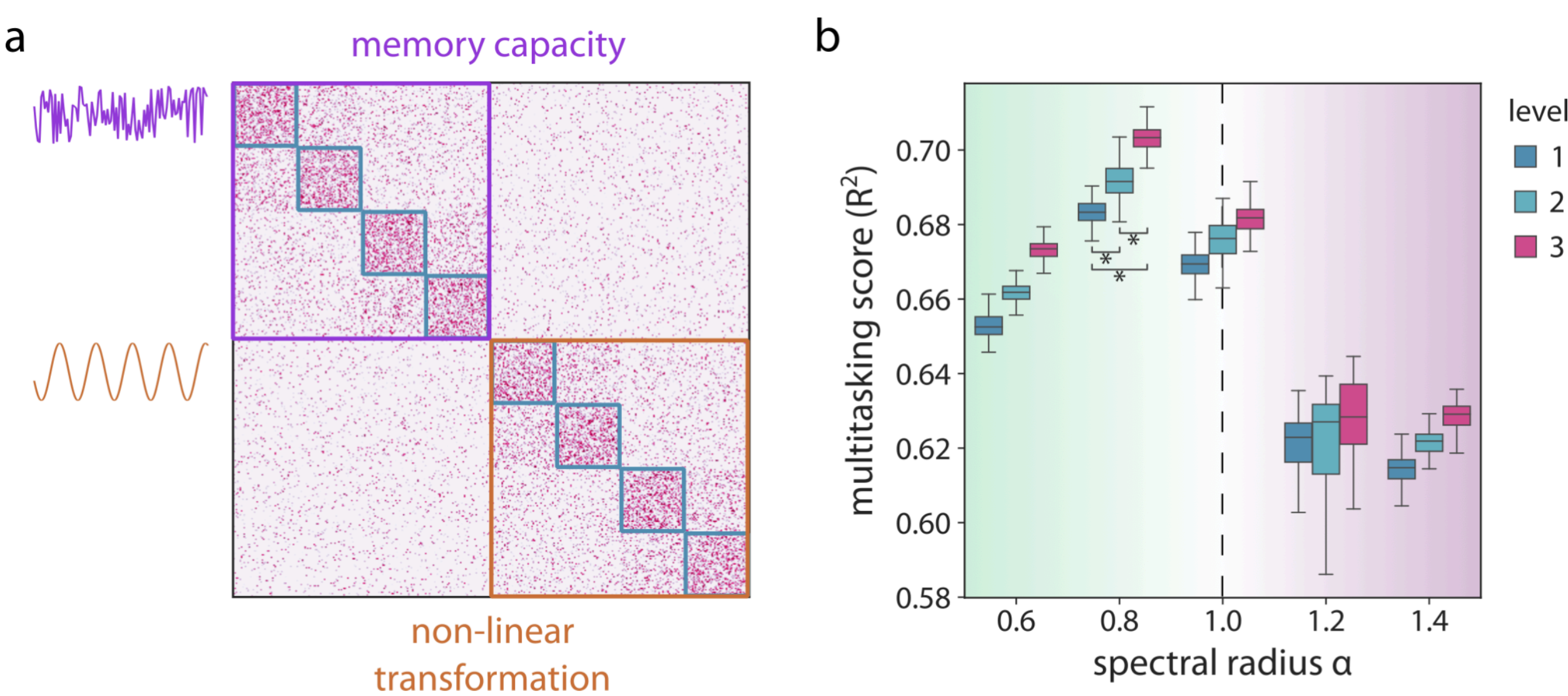

Figure 5 **Hierarchical modularity supports multitasking** | (a) Schematic of the multitasking framework: the 2 third-level modules are each assigned a different task: the memory capacity task and the non-linear transformation task. Both input signals are propagated simultaneously across the network. (b) Average R^2 regression score across tasks as a function of the α parameter for each hierarchical level. Asterisks indicate statistical significance of differences between levels according to Wilcoxon-Mann-Whitney tests ($p < 10^{-26}$). Significance is only shown for the highest performing regime.

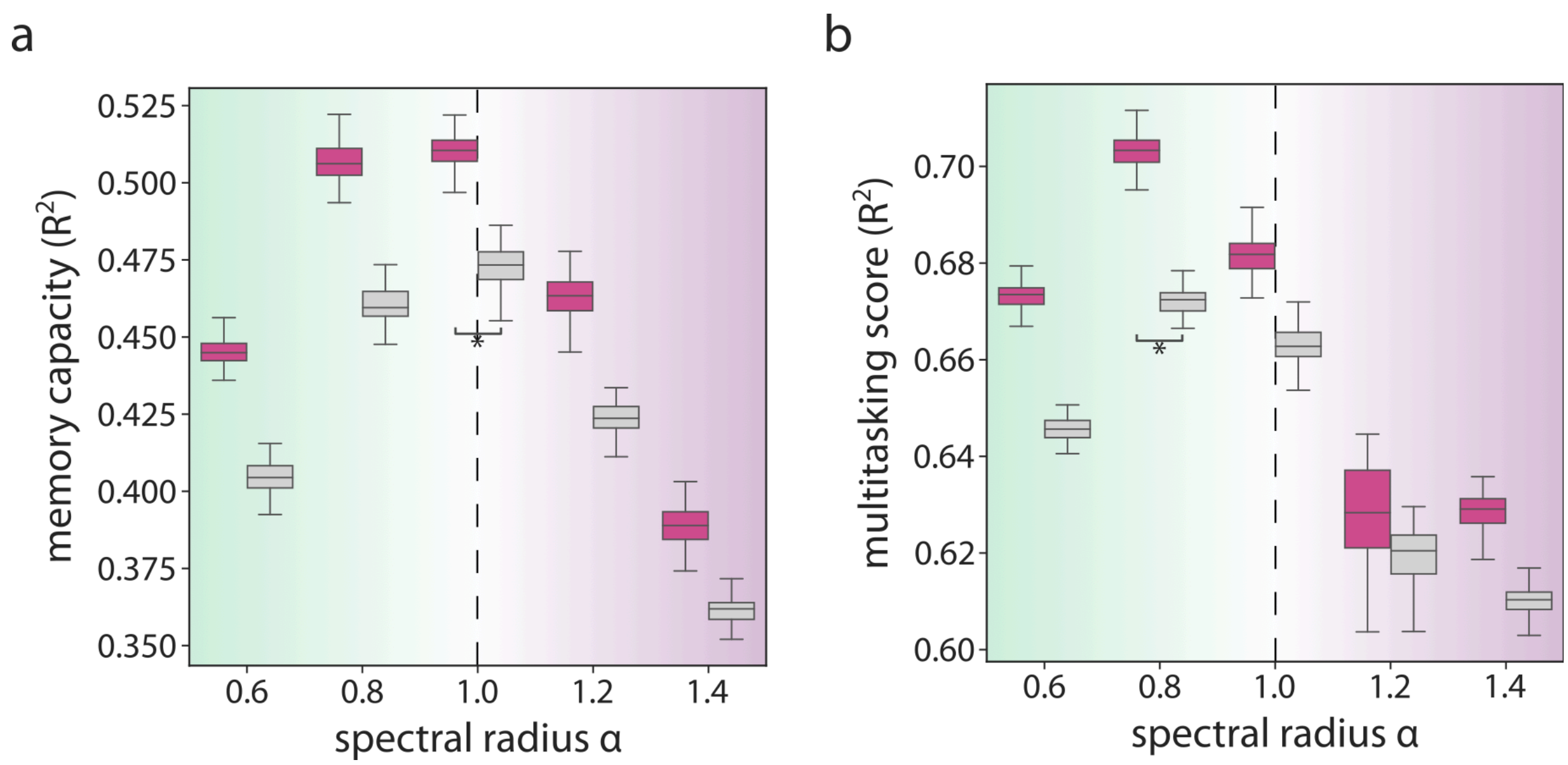

Figure S3 **Contrast between hierarchical modular and random reservoir performance** | Memory capacity (a) and multitasking score (b) as a function of the α parameter for level-3 networks (magenta) and their degree-preserving randomized surrogates (grey). Asterisks indicate statistical significance of differences between levels according to Wilcoxon-Mann-Whitney tests ($p < 10^{-33}$). Significance is only shown for the highest performing regime.

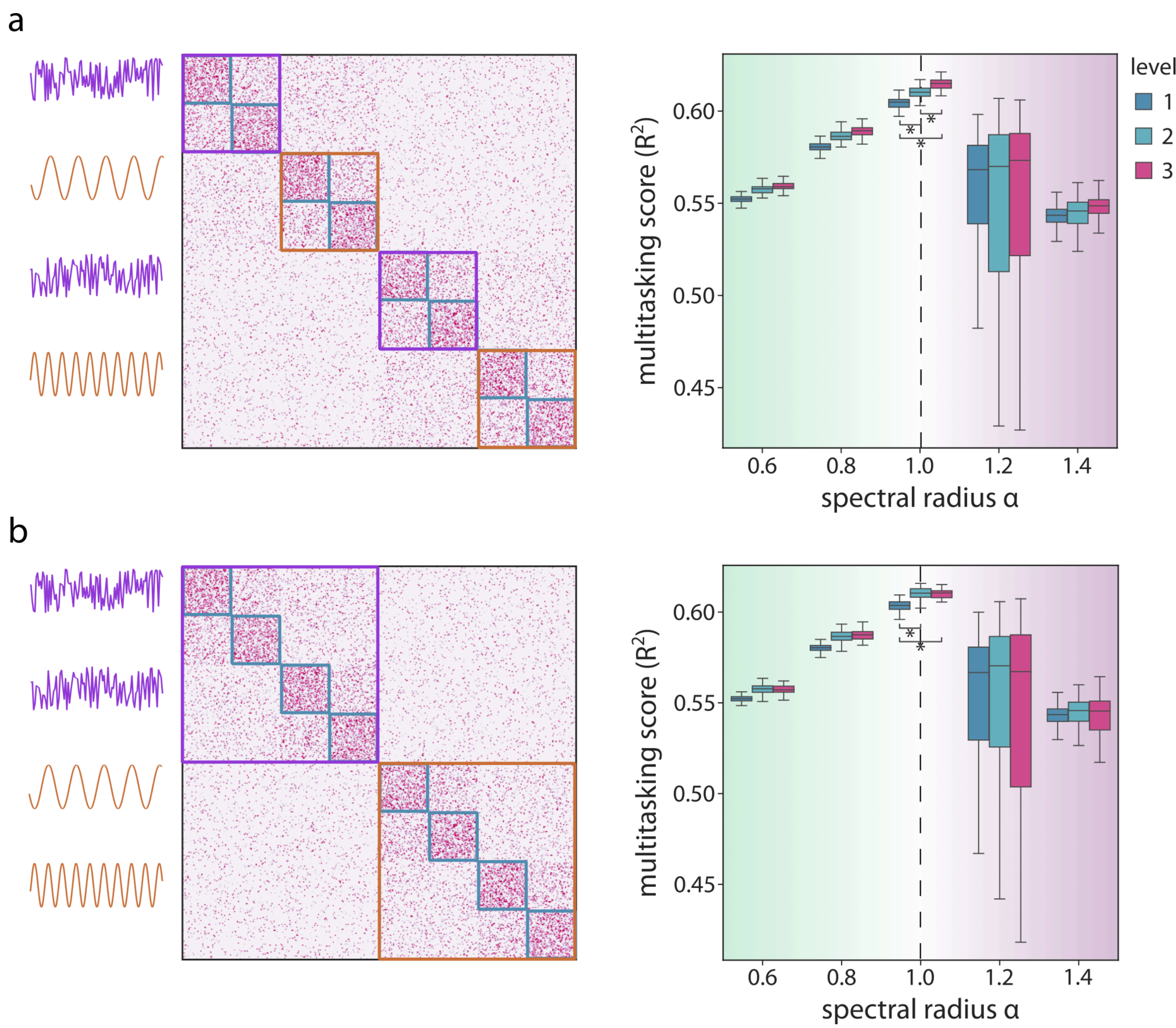

Figure S8 **Hierarchical modularity supports multitasking - 4 tasks** | (a) Left: Schematic of the multitasking framework: the 4 second-level modules are each assigned a different task. The memory capacity task and the non-linear transformation task are interleaved. Both input signals are propagated simultaneously across the network. Right: Average R^2 regression score across tasks as a function of the α parameter for each hierarchical level. (b) Same as (a) when task categories are not interleaved (the 2 third-level modules are each assigned a different task category). Asterisks indicate statistical significance of differences between levels according to Wilcoxon-Mann-Whitney tests ($p < 10^{-16}$). Significance is only shown for the highest performing regime.

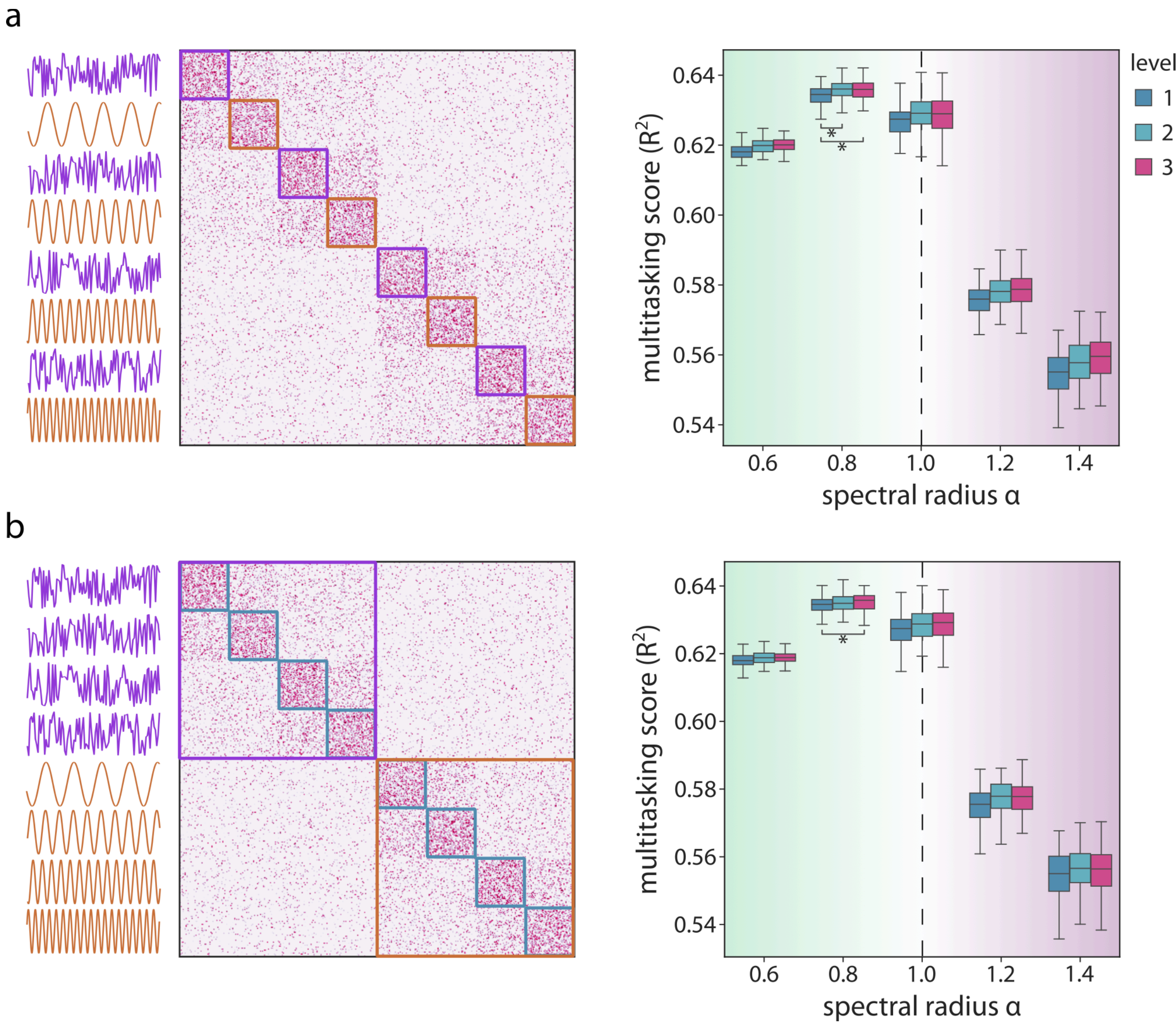

Figure S9 **Hierarchical modularity supports multitasking - 8 tasks** | (a) Left: Schematic of the multitasking framework: the 8 first-level modules are each assigned a different task. The memory capacity task and the non-linear transformation task are interleaved. Both input signals are propagated simultaneously across the network. Right: Average R^2 regression score across tasks as a function of the α parameter for each hierarchical level. (b) Same as (a) when task categories are not interleaved (the 2 third-level modules are each assigned a different task category). Asterisks indicate statistical significance of differences between levels according to Wilcoxon-Mann-Whitney tests ($p < 0.01$). Significance is only shown for the highest performing regime.

- Discussion: the overall results showing how hierarchical modularity can enhance learning and memory by expanding timescale diversity is very interesting. But the connectome results also show that topology can't explain everything and that better dynamical models (more realistic than tanh) should be considered. One suggestion I have (for future work) which would be relatively easy to implement is to consider adding internal memory to the nodes - see e.g. Verstraeten et al. An experimental unification of reservoir computing, Neural Networks, 20, 391 (2007).

We thank the Reviewer for this suggestion. In the *Discussion*, we now propose the addition of internal memory as a good first step towards examining the effect of heterogeneous dynamics:

(“Discussion” section, Paragraph #8):

> “... Future studies could examine the effect of heterogeneous dynamics, notably via node-wise internal memory (Verstraeten et al., 2007) and introduce incremental layers of biological details, including microarchitectural excitation gradients (Chaudhuri et al., 2015; Demirtaş et al., 2019), cell type-specific dynamics (Tomov et al., 2014), or geometry-informed conduction delays (Singer, 2021). ...”

- typo, p.10: motif consists in --> motif consists of

Thank you for pointing it out. We have corrected it.

Reviewer #2 (Remarks on code availability):

I did not install or run the code, but it appears to be complete and includes a README file with clear instructions, data details and additional information on external dependencies.

Thank you for the positive comments.

Dear Reviewers,

Thank you for your time and for the positive feedback on our revised manuscript. Below, we respond to the remaining concerns of Reviewer #1. Reviewer comments are in **bold font** and our responses are in regular font.

Reviewer #1 (Remarks to the Author):

The revised manuscript represents a clear and substantive improvement over the original submission. I would like to thank the authors for their thorough rebuttal and for revising the paper significantly.

The paper has been strengthened by 1. expanding the experimental scope to include more challenging benchmarks such as Lorenz and NARMA tasks, 2. providing a much more convincing mechanistic account through the introduction of cycle-preserving null models and direct perturbation experiments, which clarify the role of recurrent motifs in driving memory capacity, and 3) improving the robustness and interpretability of their analyses, particularly for neural timescales. The additional connectome-based analyses and the consideration of wiring cost tradeoffs also enhance the biological relevance of the study.

At the same time, some of my earlier concerns remain: The overall conceptual novelty is still somewhat limited relative to prior work on hierarchical and modular reservoir architectures, and the practical significance of the observed performance gains remains modest. Moreover, the results suggest that much of the benefit attributed to hierarchical modularity may in fact be explained by lower order structural features such as cycles, raising questions about how essential hierarchy itself is as a design principle. Finally, certain limitations (such as the sensitivity to the treatment of negative weights and the reliance on relatively stylized tasks) indicate that further work is needed to establish the generality and practical applicability of the findings.

We thank the Reviewer for their positive comments. We also agree that some limitations remain. We have therefore made sure to include them in the revised manuscript:

> (“Discussion” section, Paragraph #4):
>
> “… Here, we find that modular networks matched in the number of short cycles to hierarchical modular networks reach similar memory capacity. This suggests that the functional enhancement provided by hierarchical modularity

can be explained by lower-order cyclic structure. Importantly, this clarifies the mechanisms underlying the benefits of a ubiquitous network architecture and positions our framework as a parsimonious approach for introducing structured cyclicity. Furthermore, this equivalence does not extend to the multitasking setting, where hierarchical modular reservoirs outperform cycles-preserving null models, potentially because their nested organization enables principled input and output placement across modules. Altogether, these results suggest that cycles play an important role, but do not fully account for the computational advantages conferred by hierarchical modularity."

("Discussion" section, Paragraph #9):

"The present findings should be interpreted with respect to some methodological limitations. First, we focus on benchmark tasks that, while common in reservoir computing, remain relatively simple. Future work should examine whether these findings generalize to more complex tasks and real-world applications. … Third, we find that the effect of hierarchical modularity on memory capacity only generalizes to signed networks when negative weights are introduced in first-level modules. This indicates that random placement of negative weights might effectively blur a binary hierarchical modular organization. Given the importance of negative connections for representation capacity (Wang et al., 2023), future work should explore how to most efficiently integrate them in hierarchical modular architectures."
